# Advancing the clinical assessment of glomerular podocyte pathology in kidney biopsies via super-resolution microscopy and angiopoietin-like 4 staining

**DOI:** 10.7150/thno.101498

**Published:** 2025-01-01

**Authors:** Xiaojing Liu, Suxia Wang, Gang Liu, Yan Wang, Shunlai Shang, Guming Zou, Shimin Jiang, Xuliang Wang, Li Yang, Wenge Li

**Affiliations:** 1 Department of Nephrology, China-Japan Friendship Hospital (Institute of Clinical Medical Sciences), Beijing, 100029, China.; 2 China-Japan Friendship Hospital (Institute of Clinical Medical Sciences), Chinese Academy of Medical Sciences & Peking Union Medical College, Beijing, 100730, China.; 3 Laboratory of Electron Microscopy, Pathological Center, Peking University First Hospital, Beijing, 100034, China.; 4 Renal Division, Peking University Institute of Nephrology, Key Laboratory of Renal Disease-Ministry of Health of China, Peking University First Hospital, Beijing, 100034, China.; 5 Department of Nephrology, Xijing Hospital, Air Force Medical University, Xi'an, China.; 6 The Kidney Disease Center, the First Affiliated Hospital, Zhejiang University School of Medicine, Hangzhou, Zhejiang Province, 310000, China.

**Keywords:** podocytes, 3D imaging, angiopoietin-like 4, renal biopsy, super-resolution confocal microscopy

## Abstract

**Rationale:** The tertiary structure of normal podocytes prevents protein from leaking into the urine. However, observing the complexity of podocytes is challenging because of the scale differences in their three-dimensional structure and the close proximity between neighboring cells in space. In this study, we explored podocyte-secreted angiopoietin-like 4 (ANGPTL4) as a potential morphological marker via super-resolution microscopy (SRM).

**Methods and Results:** Specimens from patients with minimal change disease (MCD), focal segmental glomerulosclerosis (FSGS), and membranous nephropathy (MN), along with normal controls, were analyzed via immunofluorescence and immunohistochemistry to determine the expression and localization of ANGPTL4, confirming its extensive presence in podocytes across both healthy and diseased conditions. Immunoelectron microscopy revealed that ANGPTL4 is distributed throughout the podocyte cell body, primary processes, and foot processes. Compared with conventional podocyte markers such as nephrin and synaptopodin, ANGPTL4 excels in depicting the three-dimensional structure of podocytes via SRM imaging. We then refined a protocol using tyramide signal amplification staining and confocal microscopy to uniformly enhance podocyte fluorescence, facilitating the clinical assessment of biopsies. In patients diagnosed with MCD and FSGS, measurements of slit diaphragm density, primary process width, and foot process width were taken after further co-staining with nephrin to identify patterns of podocyte morphological alterations. Distinctive patterns of foot process effacement were identified in MCD and FSGS patients, with FSGS patients showing more pronounced podocyte injury.

**Conclusions:** ANGPTL4 serves as a reliable morphological marker for podocyte analysis, offering enhanced visualization of their three-dimensional structure and facilitating the identification of distinct pathological changes in nephrotic syndrome patients.

## Introduction

Podocytes play crucial roles in the glomerular filtration process 1, and their unique structure comprises the cell body, primary processes (PPs), and foot processes (FPs), which are interconnected by the slit diaphragm (SD) 2. FP effacement (FPE), a hallmark of glomerular diseases, is characterized by changes in the structure of FPs and SDs, leading to a reduction in the filtration surface area and impaired filtration function 3-5. Given the importance of these cellular structures in maintaining renal function, the ability to accurately assess podocyte morphology is paramount for understanding and managing kidney pathologies.

Super-resolution microscopy (SRM) has revolutionized podocyte microscopy by enabling quantitative analysis of FPE through the abundance of SDs, surpassing the constraints of traditional 2D transmission electron microscopy (TEM) images 6-11. Endlich 12 and Benzing 8, along with other research teams 8, 13, 14, have developed semiautomated and automated quantification methods for FPE based on nephrin-stained SDs. However, current markers, including synaptopodin (SYNO, an actin-associated protein in podocyte FPs) and nephrin, are limited in depicting the full podocyte structure owing to their selective expression 15, 16. Although focused ion beam scanning electron microscopy (FIB/SEM) excels in 3D podocyte ultrastructure evaluation, its destructive nature limits its clinical application 17. There is an urgent need for a marker that uniformly labels the entire podocyte to improve SRM visualization of podocyte structural changes in podocytopathy.

Angiopoietin-like 4 (ANGPTL4), a multifaceted secreted protein, gained prominence in the field of nephrology when Chugh's team reported a significant increase in both serum and podocytes in human and experimental minimal change disease (MCD) models 18. Podocytes secrete a hyposialylated form of ANGPTL4 that mediates the generation of proteinuria. Subsequent studies revealed that in untreated patients with nephrotic syndrome, fasting plasma ANGPTL4 levels significantly increased with a higher free fatty acid-to-albumin ratio, occurring not only in MCD but also in focal segmental glomerulosclerosis (FSGS) and membranous nephropathy (MN) 19. ANGPTL4 has multiple binding sites in the glomerulus, including integrin-associated β1 (podocyte α3β1) and β5 (glomerular endothelial αvβ5), and circulating ANGPTL4 can interact with these integrin receptors as well as non-integrin proteins such as syndecans and heparan sulfate, thereby affecting proteinuria 20-22.

In this study, we aimed to visualize the overall structure of podocytes in patients with primary nephrotic syndrome through immunostaining for ANGPTL4, which is either secreted by podocytes or derived from the blood circulation, thereby measuring podocyte morphology and assessing podocyte injury. We found that ANGPTL4 is significantly expressed in podocytes under various glomerular diseases and healthy conditions and then mapped the distribution of ANGPTL4 across podocyte subcompartments, confirming its superiority as a universal marker over traditional SRM markers. We further developed an enhanced ANGPTL4 staining protocol integrated with array confocal microscopy to increase sensitivity, enabling accurate 3D imaging of podocyte abnormalities and fusion. This methodology illuminates distinct FPE patterns in MCD and FSGS patients, providing a powerful tool for assessing podocyte morphology and disease pathology across a range of conditions.

## Methods

### Human and mouse kidney tissue

A total of 74 human kidney samples, including 63 renal biopsy samples, 3 donor kidney samples, and 8 normal renal tissue samples adjacent to renal tumors, were collected at three medical centers: Peking University First Hospital, China-Japan Friendship Hospital, and the First Affiliated Hospital of Zhejiang University School of Medicine. Tissue samples dissected from the nontumorous pole of the kidney presented a normal histologic picture on routine histologic examination.

Nineteen patients with MCD, eight patients with MN, five patients with primary FSGS, and five patients with normal renal tissue adjacent to renal cell carcinoma were from Peking University First Hospital. Twelve patients with MCD, nine patients with MN, seven patients with primary FSGS, three patients with tubulointerstitial nephritis (TIN), and three patients with normal renal tissue adjacent to renal cell carcinoma were from China-Japan Friendship Hospital.

Three donor kidney samples were obtained from the First Affiliated Hospital, Zhejiang University School of Medicine (Zhejiang, China). The time zero (implantation) biopsy is performed after the completion of the vascular and ureteral anastomoses during the recipient surgery (before reperfusion). Two 18-gauge core needle biopsies were obtained via an automated biopsy gun.

The study was conducted following the principles outlined in the Declaration of Helsinki. This study was approved by the Ethics Committee of China-Japan Friendship Hospital (No. 2024-KY-360).

Both male whole-body* Angptl4* knockout mice and male C57BL/6 mice, as well as male Sprague-Dawley rats, were acquired from Cyagen Biosciences (Cyagen Biosciences, Guangzhou, China). The mice were sacrificed at the age of 10 weeks, while the rats were sacrificed at 12 weeks of age. All experiments were approved by the China-Japan Friendship Hospital's Animal Research Ethics Committee (No. zryhyy21-22-10-03). No abuse or maltreatment occurred during our study.

### Immunofluorescence staining

For indirect immunofluorescence, phosphate-buffered saline (PBS) was used as a diluent for all steps. Three-micron-thick formalin-fixed paraffin-embedded (FFPE) sections were deparaffinized with dimethylbenzene and rehydrated through a graded alcohol series. Antigen retrieval was performed in Tris-EDTA buffer (20 mM Tris, 1 mM EDTA, pH 9.0) at high pressure and temperature for 2 min, followed by cooling at room temperature. The slides were then washed in PBS and blocked in goat serum at room temperature for 30 min. In all the cases, the sections were incubated with primary antibodies at 4 °C overnight and with secondary antibodies for 1 h. For double/triple immunofluorescence staining procedures, the corresponding two or three primary antibodies and their corresponding two or three secondary antibodies were simultaneously incubated. To stain for ANGPTL4, a rabbit anti-human ANGPTL4 primary antibody (ProteinTech Group, Chicago, IL, USA; catalog number, 51109-1-AP, 1:50) and a goat anti-rabbit DyLight 488-conjugated secondary antibody (Jackson ImmunoResearch, USA, 1:500) were used. To stain for SYNO, a mouse anti-SYNO primary antibody (ProteinTech Group, Chicago, IL, USA; catalog number, 66970-1-Ig, 1:2000) and an Alexa Fluor 647 donkey anti-mouse secondary antibody (Thermo Fisher Scientific, #A-31571, 1:500) were used. To stain for podocalyxin, a mouse anti-podocalyxin primary antibody (ProteinTech Group, Chicago, IL, USA; catalog number, 68250-1-Ig, 1:2000) and an Alexa Fluor 647 donkey anti-mouse secondary antibody (Thermo Fisher Scientific, #A-31571, 1:500) were used. To stain for nephrin, a polyclonal guinea pig anti-nephrin primary antibody (Progen, Heidelberg, Germany; catalog number, PG-N2, 1:150) and a goat anti-guinea pig Cy3-conjugated secondary antibody (Jackson ImmunoResearch, Hamburg, Germany; catalog number,706-165-148, 1:500) were used. DAPI (104139; Abcam) was used to stain the nuclei.

### Immunohistochemical (IHC) staining

The procedures used before the secondary antibody incubation were the same as those used for the immunofluorescence experiments mentioned above. After incubation with a horseradish peroxidase (HRP)-conjugated goat anti-rabbit secondary antibody (Zhongshan Goldenbridge Biotechnology) at room temperature for 30 min, immunodetection was carried out via DAB staining (Zhongshan Goldenbridge Biotechnology Company, Beijing, China).

Fiji software was used for image analysis to quantify the areas of glomeruli with positive ANGPTL4 staining and to assess the integrated optical density (IOD) of the images obtained. At least 3 glomeruli/section were analyzed. The relative expression of ANGPTL4 was determined by calculating the average optical density (AOD), defined as the ratio of the IOD to the area of positive staining.

### Immunofluorescence analysis of cryosections

For immunofluorescence staining of cryosections, fresh 4 μm-thick OCT-embedded tissue was used. No fixation agent was applied. The slides were then washed in PBS and blocked in goat serum at room temperature for 30 min. Primary antibodies were used for 24 h at room temperature. Secondary antibodies were used for 1 h at room temperature. To stain for ANGPTL4, a rabbit anti-human ANGPTL4 primary antibody (ProteinTech Group, Chicago, IL, USA; catalog number, 51109-1-AP, 1:50) and a goat anti-rabbit DyLight 488-conjugated secondary antibody (Jackson ImmunoResearch, USA, 1:500) were used. To stain for podocalyxin, a mouse anti-human podocalyxin antibody (Invitrogen catalog number, 393800, 1:500)) and a donkey anti-mouse DyLight 649 secondary antibody (Jackson ImmunoResearch, USA, 1:500) were used. To stain for laminin, a rat anti-human antibody against laminin β2γ1 (Abcam, 1:500) and a goat anti-rat Dy3-labeled secondary antibody (Life Technologies, 1:500) were used. To detect the nucleus, DAPI (104139; Abcam) was applied for 20 min at room temperature.

### Immunoelectron microscopy and EM

Immunoelectron microscopy of kidney biopsies was performed according to the methods of Johnson with modifications 23. For immunogold labeling, kidney biopsies (n = 2) and controls (n = 1) were successively fixed with a mixture of 4% paraformaldehyde/0.25% glutaraldehyde in 0.1 mol/L phosphate buffer, pH 7.2, for 1 h, followed by the addition of 4% paraformaldehyde, and then processed for ultracryomicrotomy. The grids were placed on 2% gelatin at 37 °C for 30 min, quenched with 50 mmol/L glycine in phosphate buffer, blocked with 1% bovine serum albumin and 10% normal goat serum, and then incubated with ANGPTL4 antibody (1:50 dilution) in phosphate buffer containing 1% bovine serum albumin and 2% normal goat serum for 2 h. The grids were washed twice, followed by incubation with goat anti-rabbit IgG coupled to 10-nm colloidal gold particles (British Biocell International, Cardiff, UK). After washing, the cryosections were stained with 2% uranyl acetate and embedded in 2% methylcellulose containing 5% uranyl acetate.

EM was conducted via an EM-100CX transmission electron microscope (JEOL, Tokyo, Japan).

### *In situ* hybridization

mRNA *in situ* hybridization was carried out manually via a PinpoRNA RNA *in situ* hybridization kit (GD Pinpoease Biotech Co. Ltd., Cat#: PIT0001; website: https://www.pinpoease.com). A series of short probes sequentially complementary to the ANGPTL4 target RNA sequence covering the 225--1640 region were designed via patented algorithms (CHINA patent number ZL202110581853.9). Briefly, the tissue was treated with pre-A solution to inhibit endogenous peroxidase activity and boiled with pre-B solution B to recover the RNA binding site. Protease treatment was then used to expose the target RNA molecules, followed by probe hybridization for 2 h at 40 °C. The signal was subsequently amplified sequentially by reactions 1, 2 and 3 at 40 °C, followed by incubation at room temperature for reactions 4 and 5 (China patent number ZL202110575231.5). Finally, a fast-red substrate is added, and the signal is shown as precipitated red dots.

### Tyramine signal amplification (TSA) for ANGPTL4 immunostaining

A TSA kit (Neon, DFT4C50, China) was utilized for signal amplification for the detection of ANGPTL4 following the manufacturer's instructions, with subsequent optimization and modifications. The heat-induced antigen retrieval process was conducted as previously described. The experiment utilized a horseradish peroxidase-labeled goat anti-rabbit secondary antibody, which was subsequently developed with a fluorescent dye diluted with the signal amplification reagent provided in the kit. The optimal incubation period for the fluorescent dye (DendronFluor®NEON570) was established at 1 min using a 1:100 dilution ratio.

For the costaining of nephrin, an indirect immunofluorescence protocol was used. Both primary antibodies were coincubated, followed by sequential application of a secondary antibody specific for nephrin and subsequent staining with a secondary antibody specific for ANGPTL4 via the TSA process.

### Imaging and super-resolution imaging

Wide-field imaging was performed via a Nikon 90i microscope (Nikon, Tokyo, Japan), and standard confocal images were acquired via a Zeiss LSM 780 microscope (Zeiss, Germany) equipped with a 100×/1.49 or on a STELLARIS 8 system equipped with a 40× objective.

Super-resolution confocal imaging was carried out via two distinct platforms: a STELLARIS 8 STED FALCON microscope (Leica Microsystems) and a Nikon AX R confocal laser scanning microscope equipped with a Nikon spatial array confocal (NSPARC) detector (Nikon Corporation, Tokyo, Japan). For the Leica microscope, all images were acquired with a 100× objective, which always implies a pinhole setting of 0.7 AU for the z-stacks in LIGHTNING mode on a STELLARIS 8 FALCON FLIM microscope. For NSPARC, images were acquired via a 100× (NA 1.49) objective and recorded with a 4096 × 4096 pixel and a pinhole of 0.2 AU when NSPARC was used, and each field of view was adjusted appropriately to cover the entire glomerulus. For 3D confocal volume, z-stacks were acquired at 0.2-0.3 μm. Subsequent image processing was accomplished via NIS-Elements AR version 4.51 (Nikon).

3D structured illumination microscopy (3D SIM) images were acquired on an N-SIM (Nikon Instruments, UK) instrument with a 100×1.49 NA lens. The step size for the Z-stacks was set to 0.20 μm. For each focal plane, 15 images (five phases, three angles) were captured with the NIS-Elements software. SIM image processing, reconstruction and analysis were carried out via the N-SIM module of the NIS-Element Advanced Research software. 3D-SIM reconstruction was performed via the following parameters: illumination modulation contrast: 1.8; high-resolution noise suppression: 2.5; out-of-focus blur suppression: 0.1-0.2.

### Immunofluorescence quantitative analysis

Confocal images were captured via the STELLARIS 8 system with a 40× objective lens. The images were acquired under uniform imaging parameters (smart gain set to 2.5 and smart intensity set to 1.26, which were determined on the basis of ensuring that the glomerulus from the normal tissue adjacent to the tumor was not overexposed). The quantification of the immunofluorescence intensity was conducted via ImageJ software. The experiments were independently replicated in the laboratory by two investigators.

### Image processing and analysis

For image analysis, ImageJ/Fiji (National Institutes of Health, Bethesda, MD) and Imaris v9.2 (Bitplane, AG) were used.

### SD index, PP width and FP width measurements

Width measurements of PPs and FPs in podocytes were carried out on ANGPTL4-labeled maximum intensity projections. The fluorescence intensities along the perpendicular edges of the PPs or branch points of the FPs were captured, and full width at half maximum (FWHM) values were extracted via the 'spiky' plugin in FIJI, thus determining the widths of these cellular structures 24. Each sample was measured at ≥ 10 sites per parameter, ensuring ≥ 2 glomeruli per slice.

The SD index, which represents the density of the slit length per unit of glomerular capillary surface area, was calculated via a previously described method 12.

### Statistical analysis

The data are expressed as the mean or mean ± standard deviation (S.D.). Measurement data were assessed with a t test or one-way ANOVA. Correlation analysis was assessed by Spearman's test. All computations were performed via GraphPad Prism software (v 9.0, GraphPad Software, Inc., San Diego, CA): *P < 0.05, **P < 0.01, ***P < 0.001, ****P < 0.0001.

### Role of funding source

The funders had no role in the study design, data collection, data analyses, interpretation or decision to submit the paper.

## Results

### Widespread detection of ANGPTL4 in podocytes across healthy and diverse glomerular pathologies

**Figure 1:**
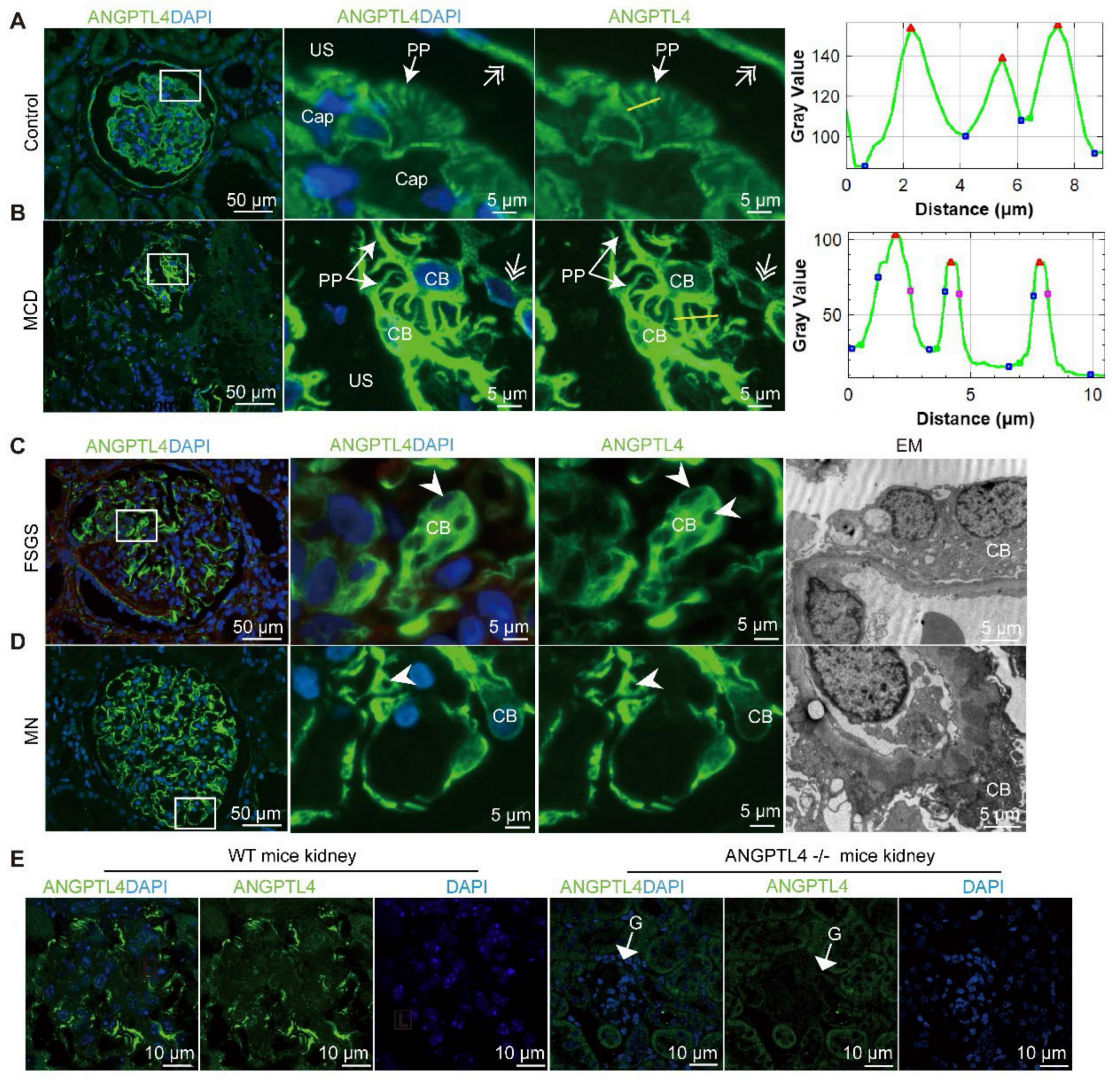
** ANGPTL4 immunostaining allows visualization of the podocyte cell body (CB) and primary processes (PPs) via conventional fluorescence microscopy (FM). (A-B)** The FM results illustrated the robust expression of ANGPTL4 within a peritumoral kidney tissue sample and in a patient with minimal change disease. (A) ANGPTL4 staining distinctly accentuates the orderly disposition of PPs and parietal epithelial cells (indicated by a double arrow). The zoomed-in images in (B) offer an en face view, where ANGPTL4 signals illustrate the juxtaposed podocyte CB and its characteristic intertwining branches. On the right-hand side of the figure, fluorescence intensity profiles are presented along two designated yellow lines, providing a quantitative understanding of the distribution of ANGPTL4 signals. Notably, the peaks within the profile corresponded to the primary processes of the podocytes. **(C)** Podocytes from a patient with focal segmental glomerulosclerosis (FSGS) display a notably enlarged and vacuolated CB (arrowhead). **(D)** Podocytes from a patient with membranous nephropathy (MN) demonstrated a loss of PPs and adhered to the capillary wall. The arrowhead points to the vacuolation of the podocytes. Scale bars = 50 μm and 5 μm (zoomed images). Representative EM images are shown in the right panel (scale bar, 5 µm). **(E)** Images of kidney sections stained with ANGPTL4 from control mice (left panel) versus* ANGPTL4* knockout mice (right panel). The glomeruli (G) of wild-type (WT) mice presented positive ANGPTL4 staining in podocytes, whereas podocyte staining was negative in knockout mice. Scale bars = 10 μm. Abbreviations: CAP, capillary lumen; US, urinary space.

To confirm the presence of ANGPTL4 in podocytes in both healthy and diseased glomeruli, we conducted immunostaining with a commercial ANGPTL4 antibody on samples from 19 MCD, 5 FSGS, and 8 MN patients and 5 normal renal tissues. The clinical details are presented in Table 1. Immunofluorescence staining revealed intense ANGPTL4 fluorescence signals within the podocytes in all the samples (Figure 1A-D). The superior signal‒to‒noise ratio enabled the discernment of podocyte morphology via conventional fluorescence microscopy. In normal controls and patients with MCD, the cell bodies and protrusions of podocytes were visible (Figure 1A-B). In stark contrast, the cell bodies in FSGS and MN patients exhibited pronounced swelling and vacuolation (Figure 1C-D).

**Figure 2:**
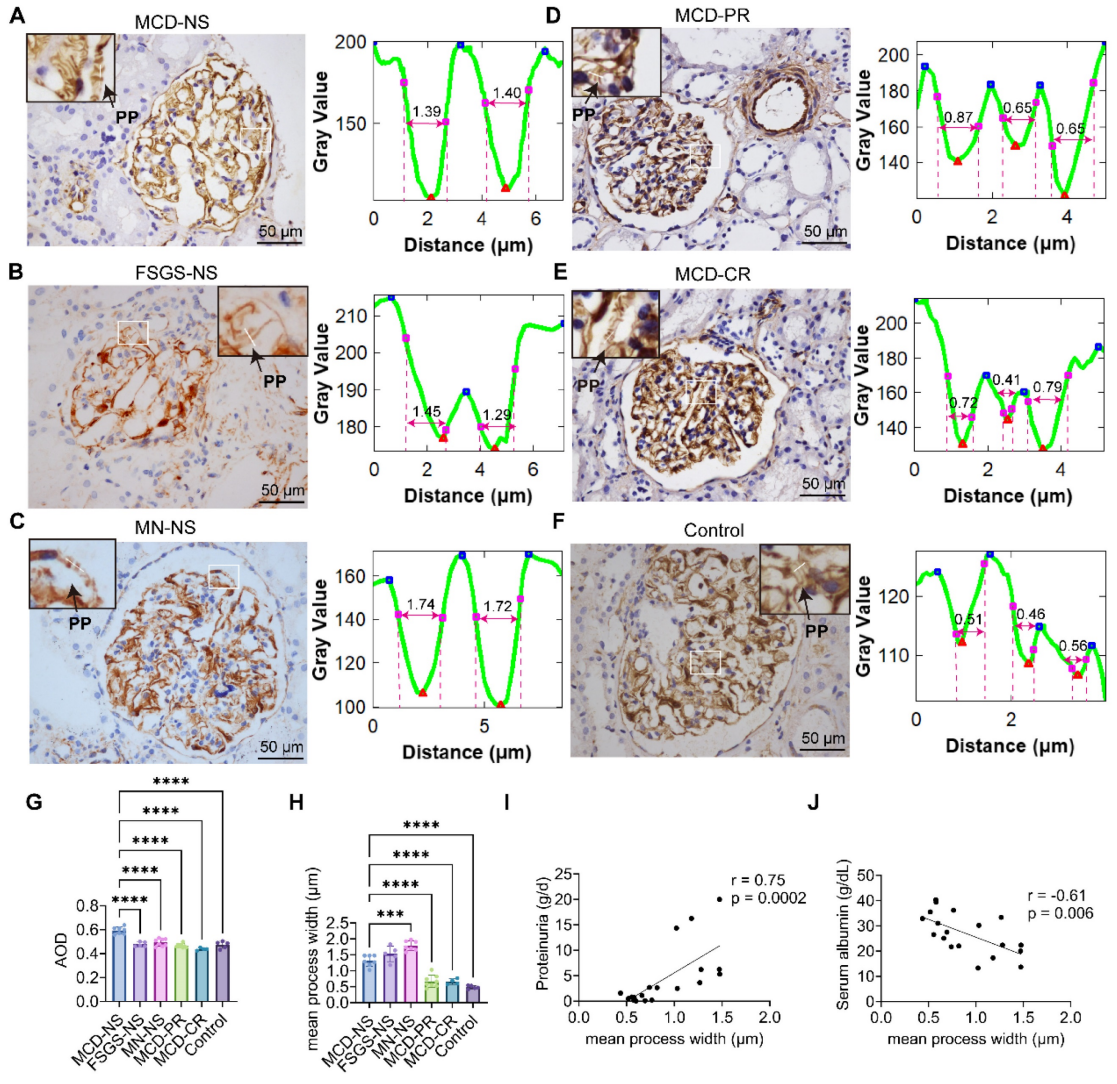
** Quantitative analysis of immunohistochemical staining and process width measurements via ANGPTL4 immunohistochemical images. (A-F)** The left panel displays ANGPTL4 immunohistochemical images with the measured area outlined (enlarged images are shown in the upper corners), and the right panel shows profile measurements of the protrusions on the capillary walls outlined from the left PANEL. The protrusion width is determined by fitting the plot profile curve via the Fiji plugin Spikey to obtain the full width at half maximum of the valley. Scale bars = 50 μm. **(G)** The protein expression of ANGPTL4 in the glomeruli was quantified by the average optical density (AOD), which was calculated by averaging over at least three glomeruli per sample.** (H)** The average process width of podocytes in each group, measured over at least ten vascular loops at three glomeruli per sample. The data are presented as the means ± standard deviations. Statistical significance was analyzed via one-way ANOVA. ***P < 0.001, ****P < 0.0001. **(I-J)** Spearman correlation analysis of the relationships between the mean process width and proteinuria, as well as between the mean process width and the level of albumin, in 19 MCD patients revealed positive and negative correlations. The Spearman correlation coefficient and the exact p value are provided. Abbreviations: CR, complete remission; NS, nephrotic syndrome; PR, partial remission; FSGS, focal segmental glomerulosclerosis; PP, primary process; MCD, minimal change disease; MN, membranous nephropathy.

To further confirm the expression levels of ANGPTL4 in podocytes across different diseases, we also conducted IHC staining (Figure 2A-F) followed by semiquantitative analysis (Figure 2G). The results revealed peak glomerular ANGPTL4 expression in the MCD nephrotic phase (n = 7), with a decline in partial (n = 8) and complete remission (n = 4); however, substantial ANGPTL4 expression remained. Patients with MN (n = 8) and FSGS (n = 5) presented lower levels of ANGPTL4 expression than did those with MCD, yet the expression of ANGPTL4 remained significantly different. Notably, normal adjacent renal tissue also exhibited notable ANGPTL4 expression (Figure 2F). The specificity of the antibody was validated by the pronounced fluorescence within podocytes of normal mouse kidney tissue (Figure 1E), normal rat kidney tissue (Figure S1), and human adipose tissue (Figure S2D), which contrasted sharply with the absence of signals in *ANGPTL4*-*KO* mouse kidneys (Figure 1E). Additionally, ANGPTL4 was distinctly expressed in renal glomerular parietal cells (Figure 1, Figure S2A-B). It is robustly expressed within the endothelial cells of small vessels, encompassing afferent and efferent arterioles (Figure S2A), as well as interstitial tissue (Figure S2C), which is consistent with prior studies 25. Notably, however, glomerular capillary endothelial cells are an exception to this pattern in biopsy samples.

Owing to the high signal‒to‒noise ratio of ANGPTL4 in podocyte protrusions, the width of these structures can be measured via light microscopy (Figure 2A‒F). The Fiji software 'spiky' plugin was used for this measurement. The results revealed that the widest protrusions, measuring 1.78 ± 0.15 μm, were found in patients with MN and were significantly swollen compared with those in patients with nephrotic MCD, with a width of 1.31 ± 0.17 μm (p < 0.001), but not significantly different from those in FSGS patients, whose protrusions measured 1.53 ± 0.24 μm (Figure 2H). Protrusions in MCD patients with partial remission (MCD-PR, 0.67 ± 0.19 μm) and complete remission (MCD-CR, 0.66 ± 0.09 μm) were significantly lower than those in the nephrotic group (1.31 ± 0.17 μm), with p values < 0.0001 for both. The thinnest protrusions were observed in adjacent normal renal tissues (0.48 ± 0.05 μm), but there was no significant difference compared with the partially and completely remitted MCD groups. When all MCD patients were analyzed collectively, there was a significant correlation between the average protrusion width and proteinuria (r = 0.75, p < 0.001; Figure 2I) as well as between the average protrusion width and the serum ALB level (r = 0.61, p < 0.01; Figure 2J), but there was no correlation with the serum creatinine level (r = 0.19, p > 0.05; Figure S2E).

These results indicate that ANGPTL4 is notably present in the podocytes of patients with various degrees of proteinuria, as well as in normal adjacent tissues, with some possibly originating from the bloodstream. Moreover, its favorable signal‒to‒noise ratio allows for the measurement of podocyte processes under a standard optical microscope, facilitating the initial clinical assessment of podocyte damage in biopsies.

### ANGPTL4 levels are markedly increased in podocytes shortly after the onset of nephrotic syndrome

To further elucidate the relationship between the expression levels of ANGPTL4 and the duration from disease onset (edema) to renal biopsy and to confirm the widespread expression of ANGPTL4 in podocytes in glomerular diseases with different renal pathologies, we included a cohort of 31 individuals from another center, comprising untreated nephrotic syndrome patients with MCD (n = 9), MN (n = 9), and FSGS (n = 4). Patients were categorized on the basis of whether the time from relapse or initial onset to renal biopsy was no more than one month (n = 12) or more than one month (n = 10); there were no significant differences in proteinuria levels or creatinine levels between the two groups (Figure S3A). Considering the potential influence of cytokine storms on ANGPTL4 expression in peritumoral kidney tissues reported by Del Nogal Avila M *et al.* 26, we expanded the control group to include patients with interstitial nephritis (n = 3) and three additional donor kidney tissues, in addition to normal peritumoral kidney tissues (n = 3). The clinical data are presented in Table 2.

**Figure 3:**
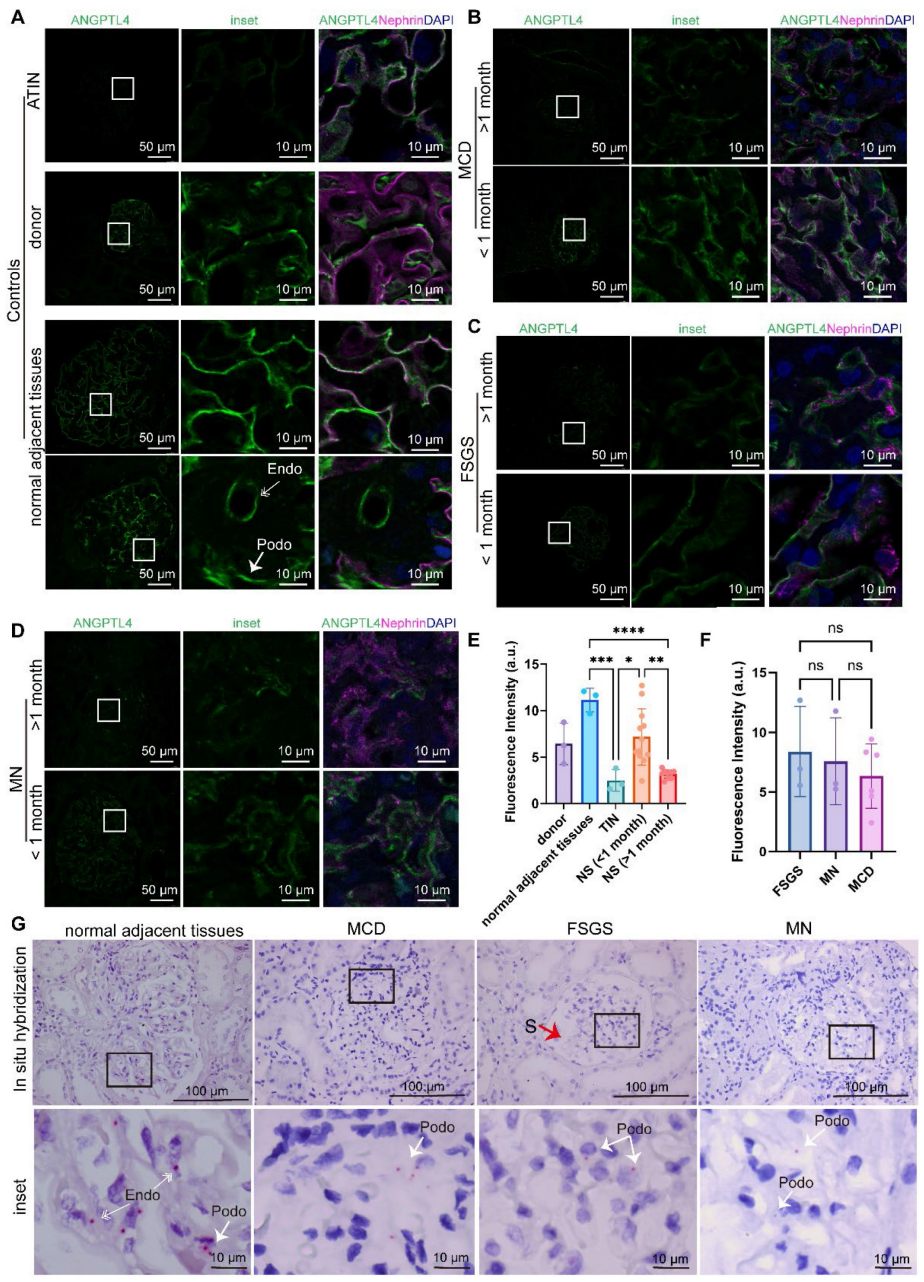
** Comparative quantitative analysis of ANGPTL4 immunofluorescence and *in situ* hybridization in patients with nephrotic syndrome biopsied at different times after relapse. (A-D)** All images were captured via a 40x objective lens on a Leica STELLARIS 8. The left panel used the same acquisition parameters (smart gain set to 2.5, smart intensity to 1.26, determined on the basis of the glomerulus from tissue adjacent to the tumor not being overexposed). The middle panel shows enlarged details of the selected images from the left panel with the same parameters. The right panel shows merged images of ANGPTL4 with DyLight 488 (green), nephrin with Cy3, and DAPI (blue) to illustrate the location of ANGPTL4 expression (exposure parameters were adjusted according to different samples). Scale bars = 50 μm for the main images and 10 μm for the enlarged images. **(E)** Quantitative results of glomerular immunofluorescence analysis across different groups, calculated by averaging over at least three glomeruli per sample. The data are presented as the means ± standard deviations. Statistical significance was analyzed via one-way ANOVA. *P < 0.05, **P < 0.01, ***P < 0.001, ****P < 0.0001. **(F)** There was no statistically significant difference in the mean ANGPTL4 fluorescence intensity among the MCD, FSGS, and MN groups who underwent biopsy less than one month after relapse. **(G)** Results of ANGPTL4 *in situ* hybridization in biopsy samples taken within one month after relapse via monochromatic chemical red staining. The arrows indicate positive areas. Scale bars = 100 μm for the main images and 10 μm for the enlarged images. Abbreviations: FSGS, focal segmental glomerulosclerosis; MCD, minimal change disease; MN, membranous nephropathy; TIN, tubulointerstitial nephritis; Endo, endothelial cell; Podo, podocyte.

We performed ANGPTL4 immunofluorescence staining on all renal paraffin sections and compared the quantitative analysis of immunofluorescence in the glomerular region across different groups (Figure 3 A-E). Notably, among the three control groups, normal peritumoral kidney tissues presented the highest expression of ANGPTL4, nearly double that of donor kidneys (p > 0.05) and over five times greater than that of TINs (p < 0.001). We observed an increase in podocyte ANGPTL4 expression in renal tissues from MCD, FSGS, and MN patients who underwent renal biopsy within one month of disease onset compared with those biopsied after one month, with fluorescence quantification approximately double that of the latter (p < 0.01). Among the five groups, patients with interstitial nephritis presented the lowest podocyte ANGPTL4 expression, which may align with the findings reported by Clement *et al.* that ANGPTL4 is present in podocytes of renal tissues at a certain level of low expression 18, 19. There was no statistically significant difference in ANGPTL4 mean fluorescence intensity (Figure 3F) or urinary protein quantification (Figure S3C) among the MCD, FSGS, and MN groups who underwent biopsies less than one month after relapse. We also compared ANGPTL4 expression between different sexes and found no significant differences (Figure S3B).

To determine the site of ANGPTL4 expression, we performed costaining with nephrin and found that in normal peritumoral kidneys, some glomerular endothelial cells also expressed ANGPTL4 (Figure 3A), which may explain the elevated expression of ANGPTL4 in peritumoral kidneys. Additionally, to delineate the cellular source of ANGPTL4, we conducted *in situ* hybridization analysis using human ppib as a positive control and bacterial DapB as a negative control (Figure S3D). The results revealed that in peritumoral kidneys, ANGPTL4 genes are expressed in both podocytes and endothelial cells, whereas in MCD, FSGS, and MN patients who underwent renal biopsy within one month of disease onset, ANGPTL4 was found only in podocytes (Figure 3G). This finding is consistent with the results of the immunofluorescence staining.

To verify the FFPE findings and explore the subcellular localization of ANGPTL4, we conducted triple immunofluorescence and immunoelectron microscopy on fresh samples (Figure S4). Three frozen sections were obtained: one from a peritumoral renal tissue sample (Figure S4A) and two from biopsies of nephrotic syndrome patients—one with MCD (Figure S4B) and one with stage 1 MN (Figure S4C). These sections were labeled for ANGPTL4, laminin (a component of the glomerular basement membrane), and podocalyxin (a transmembrane protein found on podocyte apical surfaces) and then assessed via confocal microscopy 27, 28.

Notably, these three markers consistently displayed a distinctive 'sandwich-like' arrangement (podocalyxin-ANGPTL4-laminin) juxtaposed against capillary wall structures across all three samples. Immunoelectron microscopy of the three samples further revealed that colloidal gold particles, which label ANGPTL4, were uniformly present across the podocyte substructures, including the cell body and primary/foot processes. When performing quantitative analysis of ANGPTL4 expression, it is important to exercise caution with the use of frozen sections, as the secretory protein ANGPTL4 is more prone to degradation than the structural protein laminin (Figure S5), thus requiring a meticulous comparison.

Collectively, these results further confirm the widespread presence of ANGPTL4 in renal tissues, particularly a significant increase in ANGPTL4 secretion during the early stages of nephrotic syndrome, which rapidly decreases in later stages of the disease but maintains a certain basal level of expression.

### Super-resolution mapping of ANGPTL4 in the podocyte structure

**Figure 4:**
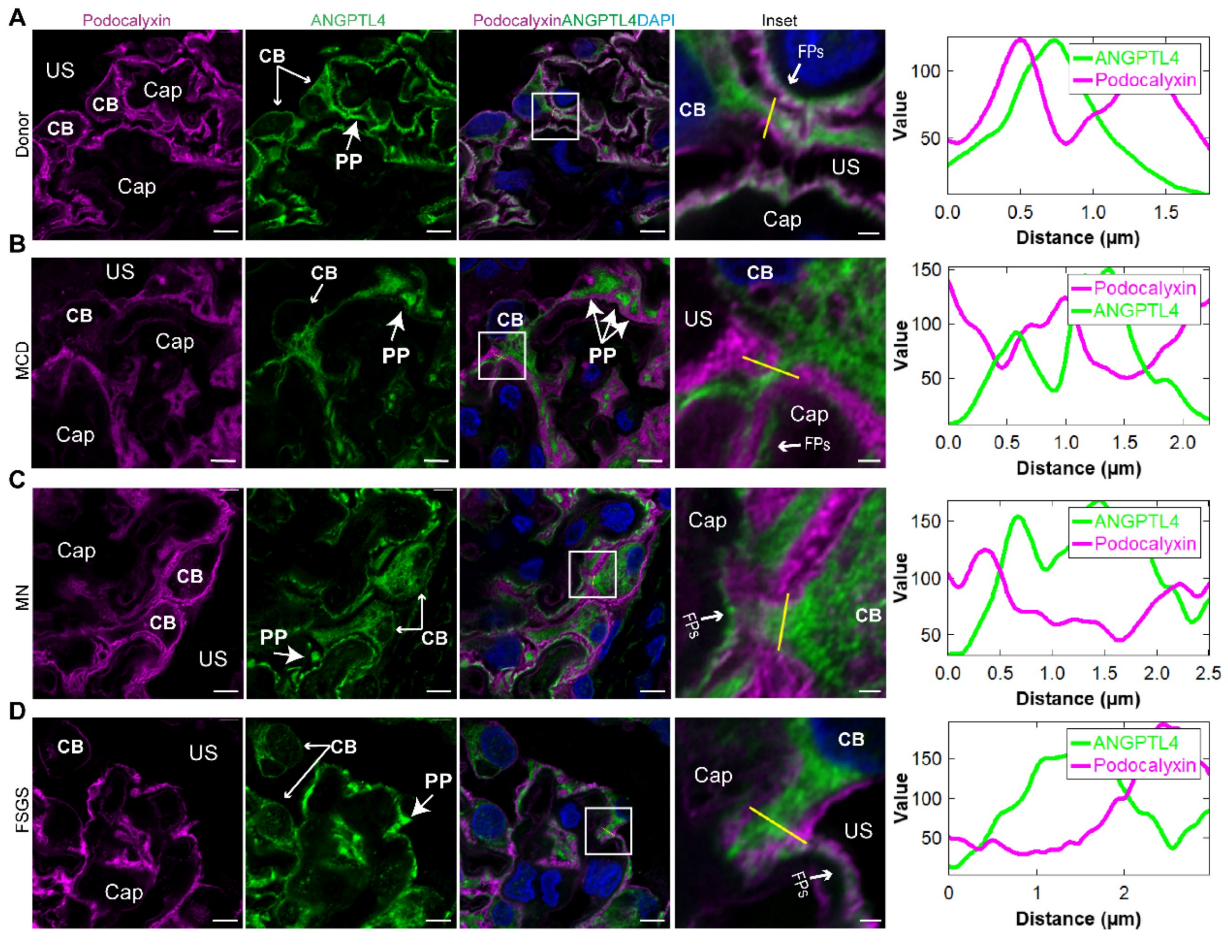
** Super-resolution images of immunofluorescence costaining for ANGPTL4 and podocalyxin from nephrotic syndrome patients and donor controls. (A-D)** All the images were captured and processed via an Airyscan system with a 100×/1.4 numerical aperture oil immersion objective lens. Three-micron-thick sections of donor kidney tissue** (A)**, MCD patient **(B)**, MN patient** (C)**, and FSGS patient **(D)** were immunostained for ANGPTL4 with DyLight 488 (green) and podocalyxin with Alexa Fluor 647 (magenta) and counterstained with DAPI (blue). Scale bars = 5 μm, with insets at 1 μm. The far right panel shows the plot profile of the outlined area in the detailed enlargement on the left. Abbreviations: FSGS, focal segmental glomerulosclerosis; MCD, minimal change disease; MN, membranous nephropathy; CAP, capillary lumen; US, urinary space; CB, cell body; PP, primary process; FP, foot process.

To achieve increased resolution of ANGPTL4 subcellular localization, we performed costaining of ANGPTL4 and podocalyxin on paraffin sections from patients with MCD, FSGS, and MN, as well as from donor kidney samples, and conducted super-resolution observations via Airyscan (Figure 4 A-D). Super-resolution confocal imaging indicated that in the cell body and primary processes, the ANGPTL4 signal was enveloped by the membrane signal of podocalyxin. On the side of the capillary lumen, ANGPTL4 is located inside podocalyxins, suggesting that ANGPTL4 is distributed across three subcompartments of podocytes—the cell body, primary processes, and FPs—indicating its potential as a marker for visualizing the three-dimensional structure of podocytes.

**Figure 5:**
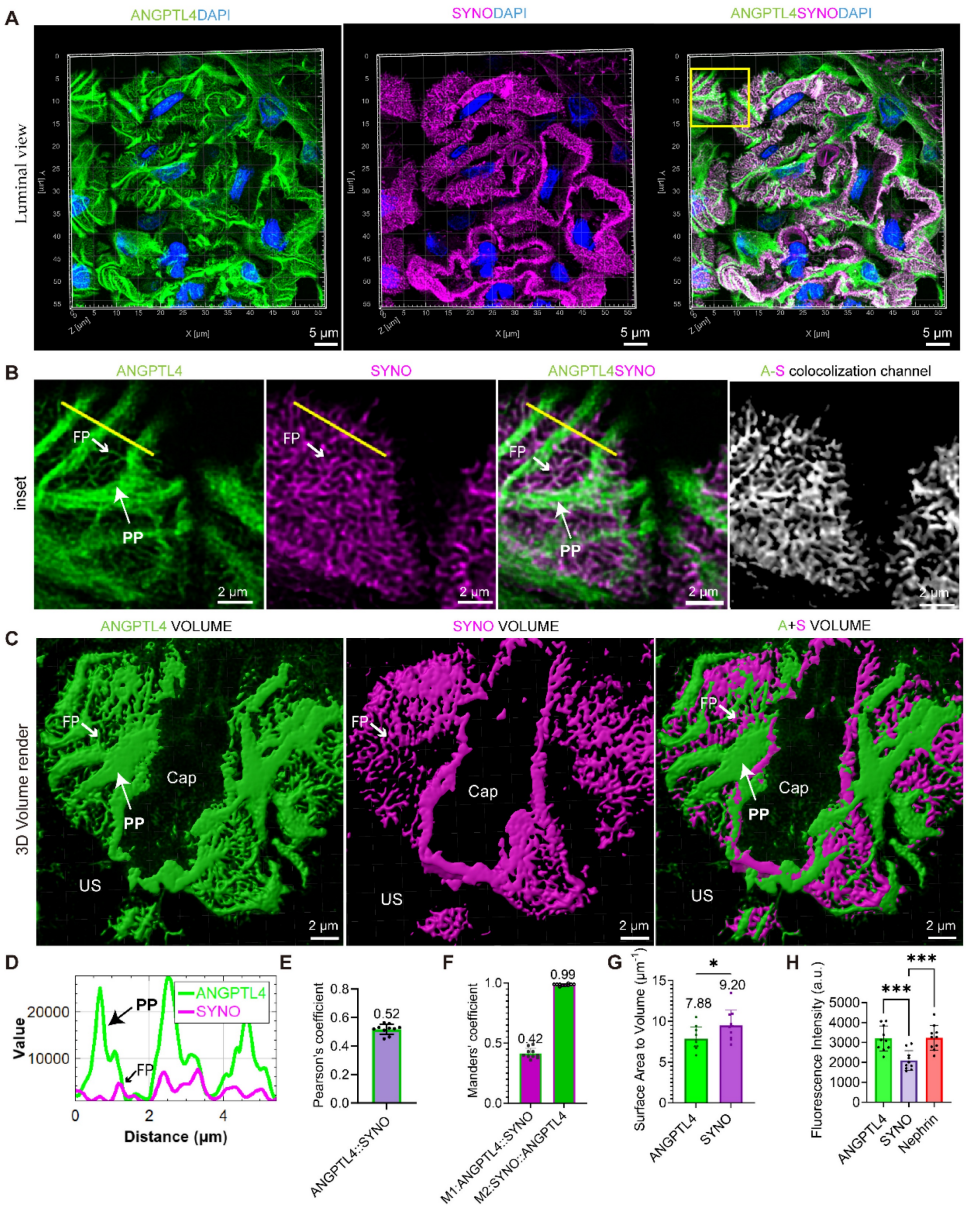
** 3D visualization of ANGPTL4 expression in podocytes derived from a donor kidney using a Leica STELLARIS 8 confocal microscope.** All images were captured and processed via a STELLARIS 8 FALCON system equipped with a 100×/1.4 numerical aperture oil immersion objective lens (pinhole set at 0.7 Airy Units). Three-micron-thick sections of donor kidney tissue were immunostained for ANGPTL4 with DyLight 488 (green) and synaptopodin (SYNO) with Alexa Fluor 647 (magenta) and counterstained with DAPI (blue). **(A)** The top row displays images showing ANGPTL4 staining, SYNO staining, and merged images. Scale bars = 5 μm. The mean Pearson's correlation coefficient for ANGPTL4 and SYNO is depicted in **(E)**, with the M1 and M2 values presented in** (F)**. **(B)** The middle panel presents enlarged views of the area within the square, with the rightmost image representing a new channel generated from the colocalization signals and the colocalization channel. Scale bars = 2 μm.** (D)** The fluorescence intensity profile along the yellow line reveals significant signal peaks for ANGPTL4 in both the primary and foot processes, whereas SYNO exhibits a peak solely in the foot processes. **(C)** The bottom row features 3D volume renderings achieved through Imaris software, depicting ANGPTL4 and SYNO volumes, respectively, indicating the overall podocyte volume and foot process volume. Scale bars = 2 μm. The podocyte and foot process surface area‒to‒volume ratios, determined from ANGPTL4 signal intensities and SYNO, are presented in **(G)**. **(H)** Immunofluorescence intensity analysis of the proteins ANGPTL4, SYNO, and nephrin. The data are presented as the means ± standard deviations. Statistical significance was analyzed via one-way ANOVA. *P < 0.05, ***P < 0.001. These data are derived from individual measurements taken from at least three separate glomeruli originating from two control samples. Abbreviations: CAP, capillary lumen; US, urinary space; CB, cell body; PP, primary process; FP, foot process.

To map the nanoscale pattern of ANGPTL4 in podocytes, STELLARIS images were captured via a STELLARIS 8 system, and postprocessing was conducted with LAS X LIGHTNING, achieving a resolution of 90 nm, which is less than half the width of the podocyte foot processes (approximately 200 nm) 29. For comparison, we examined donor kidney sections that were stained for SYNO and nephrin, in addition to ANGPTL4. These sections were analyzed via 0.2 μm Z-stacks captured with a high-resolution 100×/1.49 NA oil-immersion lens. For a comprehensive analysis of the spatial arrangement of the biomarkers, we examined them from luminal (Figure 5), cross-sectional, and basal (Figure S6) views. Luminal 3D analysis of ANGPTL4 (Figure 5A-B) revealed the podocyte architecture, including cell bodies, robust PPs, and delicate FPs. Notably, we categorized PPs into branches after the first division without differentiating further into secondary processes.

Line intensity profiling revealed notable colocalization of ANGPTL4 and SYNO in FPs (Figure 5D), with a Pearson coefficient of 0.52 (Figure 5E). Figure 5F illustrates the near-perfect colocalization of SYNO and a 42% overlap for ANGPTL4. The exceptional signal‒to‒noise ratios for ANGPTL4 and SYNO (Figure 5C) facilitated the approximate measurement of the 3D volume and surface area of podocytes and FPs. This analysis yielded an average surface-to-volume ratio of 7.88 for podocytes and 9.20 for FPs, as presented in Figure 5G.

Figure S6A shows cross-sectional views of FPs marked by ANGPTL4 and SYNO protruding from PPs and occasionally emerging below the cell body. 3D imaging revealed SYNO-enriched bridges linking nearby FPs within a single capillary and between capillaries. Corresponding structures are observed in the TEM images. Basal views (Figure S6B) showing the distribution of markers in the glomerular filtration barrier. ANGPTL4 fills large gaps in the SD network, termed ridge-like prominences (RLPs) 17, and SDs wrap around FPs with both ANGPTL4 and SYNO labels. The immunofluorescence intensities of the three proteins were compared on maximum-intensity projection images, revealing that the intensities of ANGPTL4 and nephrin were similar, suggesting relatively high expression of ANGPTL4 in the donor kidney tissue, approximately 1.5 times greater than that of SYNO (Figure 5H). This may be attributed to primary processes. We also employed SIM technology but did not achieve optimal visualization of the tertiary structures of podocytes, mainly because of structural discontinuities (Figure S7). Despite this, the technology effectively provided 2D information on podocyte FPs from single-layer images (Figure S8).

The super-resolution analysis of ANGPTL4 in podocytes confirmed its widespread distribution within podocyte subcompartments and its colocalization with SYNO, providing approximate measurements of podocyte 3D volume and surface area.

### Refining the podocyte substructure via TSA-enhanced ANGPTL4 detection

**Figure 6:**
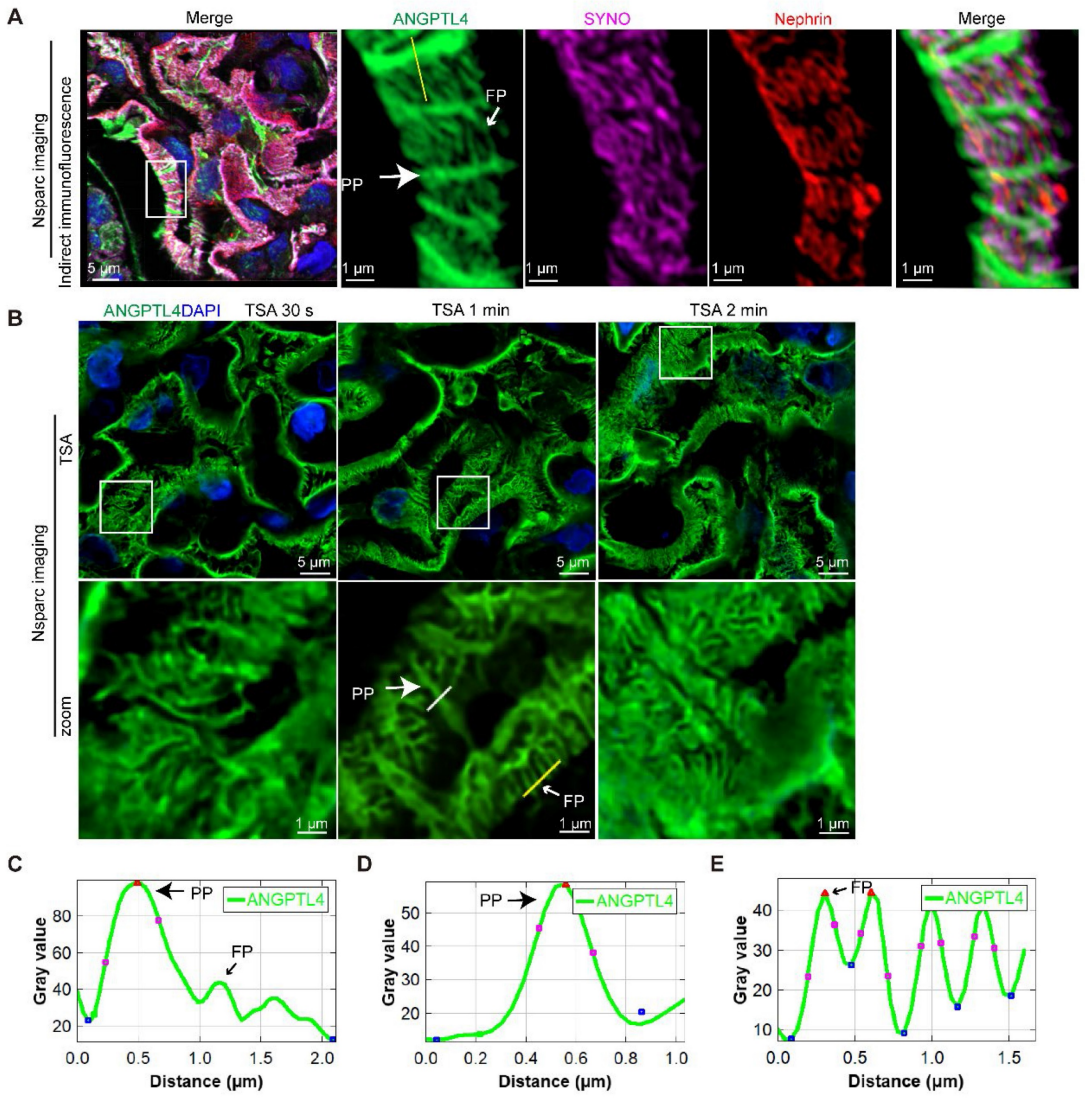
** Arrayed confocal microscopy observations of normal control kidney tissue samples immunostained for ANGPTL4 via indirect immunofluorescence versus tyramide signal amplification (TSA) techniques for comparative imaging.** All images were obtained via an Nsparc confocal microscope with an oil immersion objective at ×100 magnification and an NA of 1.49. **(A)** Indirect immunofluorescence staining of control tissues, where sections were immunostained for ANGPTL4 using DyLight 488 (green), synaptopodin (SYNO) with Alexa Fluor 647 (magenta), and nephrin with CY3 (red) and counterstained with DAPI (blue). The accompanying enlarged details illustrate that, viewed from the luminal aspect, ANGPTL4 can be discerned within the primary podocyte processes and colocalized with SYNO in the foot processes, all of which are enveloped by the meandering pattern of nephrin staining. **(B)** Representative comparative images of control renal tissues stained with a distinct dye (DendronFluor®NEON570) and incubated for 30 s, 1 min, or 2 min. The results indicate that an incubation time of 1 min resulted in a favorable signal‒to‒noise ratio and relatively uniform signal peak intensity between the primary processes and foot processes. **(C)** and **(D)**, **(E)** show the fluorescence intensity along the yellow line in A and B, respectively. C shows that the ANGPTL4 signal at foot processes is one-third of the primary process peak intensity. The foot process signals **(C)** are nearly equivalent in intensity to those of the primary processes **(D)**. Scale bars = 5 μm and 1 μm (zoomed images). Abbreviations: PP, primary process; FP, foot process.

Combining SRM with ANGPTL4 staining offers a detailed view of podocyte structure, which is useful for broader morphological assessments and as a supplementary diagnostic tool in electron microscopy for proteinuric patients when glomeruli are unavailable. To broaden the clinical utility of ANGPTL4 as a morphological marker for podocytes, we investigated more accessible array confocal microscopy methods. Popular options include the Zeiss Airyscan and the NSPARC system 30. We selected the NSPARC system for our experiments because of its accessibility. It features a 25-detector array offering 120 nm resolution in the XY plane. Z-stacks were captured at 0.2 μm intervals with a 100×/1.49 NA oil-immersion lens (Figure 6A). NSPARC microscopy distinctly revealed ANGPTL4 in podocyte PPs and FPs. However, akin to the LIGHTNING microscopy results (Figure 5D, plot profile), FPs presented a lower fluorescence intensity than did PPs (Figure 6C). To address this discrepancy, we employed TSA (Figure 6B) and fine-tuned the staining duration from 30 s to one min to achieve a consistent signal across podocyte structures with an increased signal-to-noise ratio (Figure 6D-E). In the control group, the average width of the PP was 0.47 ± 0.12 μm, which was approximately twice that of the FPs at 0.23 ± 0.03 μm, and the SD density was 3.12 ± 0.23 μm/μm², as shown in Figure 5F.

Thus, via array confocal microscopy coupled with TSA technology, we enhanced the utility of ANGPTL4 as a podocyte marker, ensuring uniform signal enhancement and facilitating the precise evaluation of alterations in podocyte tertiary structures.

### Podocyte simplification is more pronounced in FSGS patients than in MCD patients

**Figure 7:**
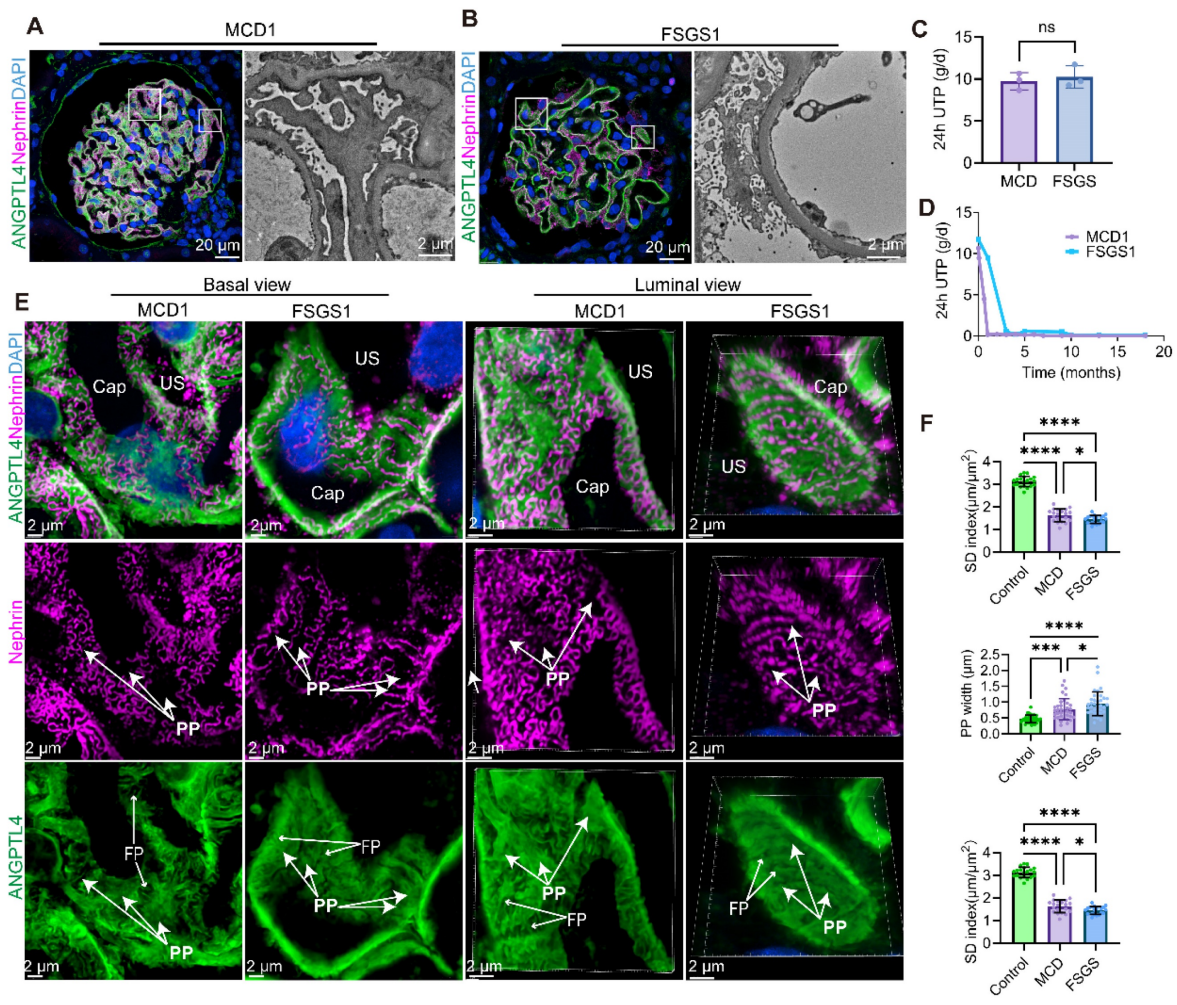
** Comparative analysis of super-resolution confocal imaging in minimal change disease (MCD) and focal segmental glomerulosclerosis (FSGS) samples costained for ANGPTL4 via tyramide signal amplification (TSA) techniques and nephrin.** Images were captured via an Nsparc confocal microscope with a ×100 oil immersion objective and an NA of 1.49. ANGPTL4 was immunostained in tissue sections with DendronFluor®NEON570 (green), stained with CY3-labeled nephrin (magenta), and counterstained with DAPI (blue). Super-resolution confocal microscopy and corresponding electron microscopy (EM) images of glomeruli from MCD **(A)** and FSGS **(B)** patients; in FSGS patients, nonsclerotic glomeruli are shown. Scale bars = 20 μm and 2 μm (EM). **(C)** Three MCD and three FSGS patients all had severe proteinuria (> 8 g/d) without intergroup differences in 24-h protein excretion. Both exhibited good responses to corticosteroid treatment **(D). (E)** MCD and FSGS glomeruli were compared via basal view maximum intensity projections and 3D luminal views. **(F)** Bar charts showing the SD density, primary process width (PP), and foot process width (FP) for controls (n = 2), MCD patients (n = 3), and FSGS patients (n = 3). At least two glomeruli per patient were measured, with ≥10 positions assessed in each glomerulus, excluding sclerotic regions, in FSGS patients. n.s., not significant, ***Significant difference (P < 0.001) between the 2 groups according to Student t test or one-way ANOVA when three groups were compared. Scale bars = 2 μm. Abbreviations: CAP, capillary lumen; US, urinary space; PP, primary process; FP, foot process.

To assess the ability of this method to visualize tertiary structural changes in podocytes during podocyte injury, we examined 3D podocyte alterations (Figure 7) in patients with MCD (n = 3) and FSGS (n = 3), all of whom exhibited severe proteinuria (Figure 7C), were treated without glucocorticoids or immunosuppressants and showed no significant difference in proteinuria. The baseline characteristics are summarized in Table 2. Damage to podocytes and the slit membrane was assessed through tissue sections labeled with ANGPTL4 via TSA amplification, followed by costaining with nephrin and imaging with the NSPARC system (Figure 7A-B).

Upon examining podocytes from MCD and FSGS patients, we examined the architecture of PPs, FPs, and SDs from luminal and basal viewpoints (Figure 7E). Notably, both MCD1 (Movie S1) and FSGS1 (Movie S1) patients showed favorable responses to steroid therapy (Figure 7D). Despite this, we observed that podocytes in both diseases displayed swelling and flattening, with FSGS patients exhibiting more severe FPE. The SD intensity and dimensions of the podocyte PPs and FPs were quantified and are presented in Figure 7F.

Patients with FSGS presented a greater than 2-fold reduction in the SD index (1.45 ± 0.17 μm/μm^2^) compared with the normal control group (3.12 ± 0.23 μm/μm^2^, p < 0.0001), an almost 2-fold increase in the primary process width (0.95 ± 0.37 μm vs. 0.47 ± 0.12 μm, p < 0.0001), and a significant widening of the FPs (0.33 ± 0.06 μm vs. 0.23 ± 0.03 μm, p < 0.0001) by approximately 1.5 times. In comparison, the MCD patients demonstrated a significant but less pronounced reduction in the SD index (1.63 ± 0.28 μm/μm^2^), which was less than half that of the normal controls. While both the primary process width (0.77 ± 0.34 μm vs. 0.47 ± 0.12 μm, p = 0.0001) and FP width (0.28 ± 0.06 μm vs. 0.23 ± 0.03 μm, p < 0.0001) increased in the MCD group, the swelling was more pronounced in the mean PP width, which exceeded that of the normal control group by more than 1.5 times. These findings indicate that FSGS elicits more severe podocyte structural changes than MCD does. These three indicators exhibit synchronized changes, yet podocyte simplification is more substantially affected by the widening of podocyte PPs in both FSGS and MCD patients.

**Figure 8:**
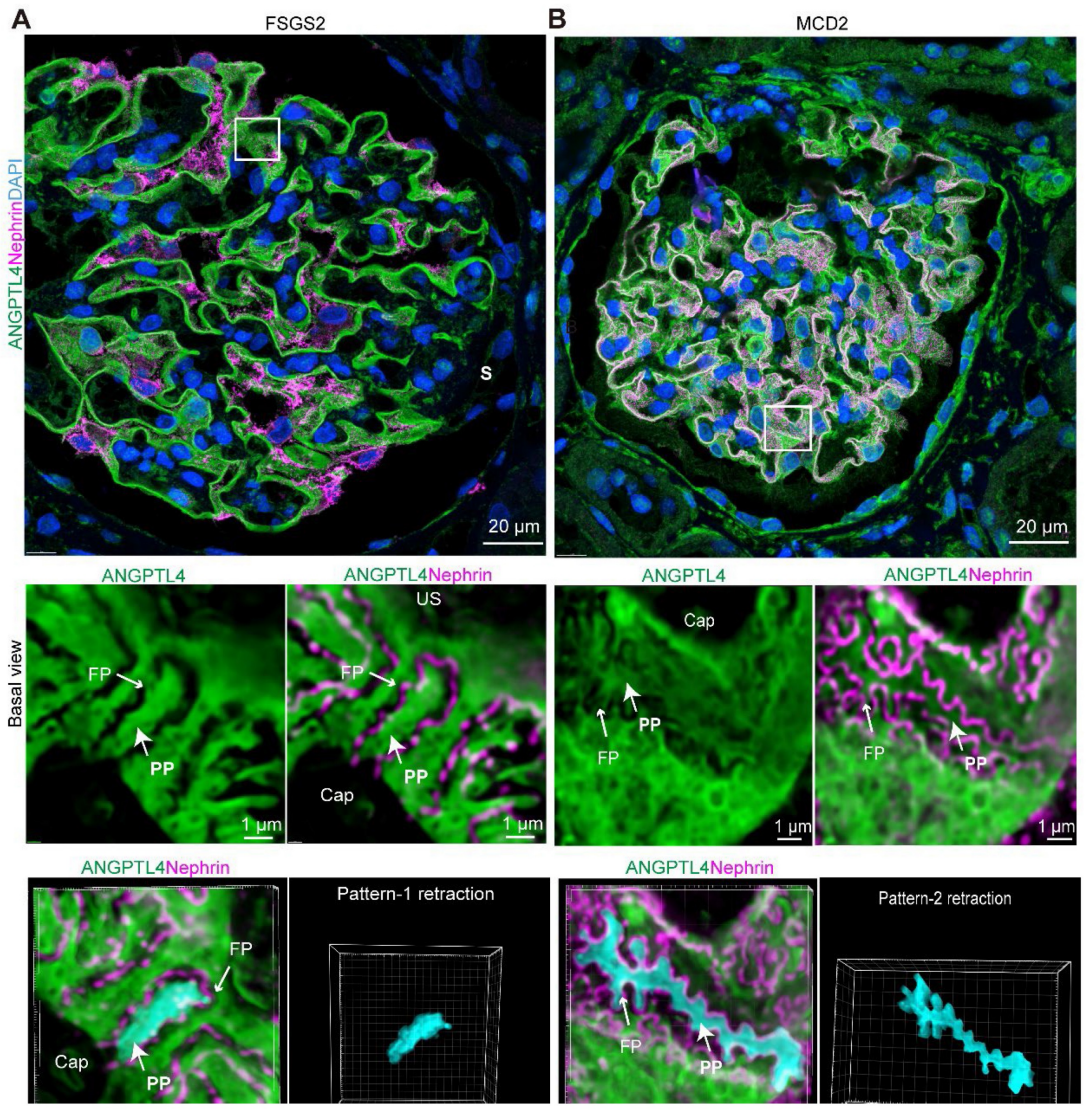
** Distinct podocyte foot process effacement in minimal change disease (MCD) and focal segmental glomerulosclerosis (FSGS) patients.** Imaging was performed via a super-resolution Nsparc confocal microscope (×100 oil objective, NA 1.49). Sections were stained for ANGPTL4 (green, DendronFluor®NEON570), nephrin (magenta, CY3), and nuclei (blue, DAPI). (A) In FSGS patients, podocyte primary processes exhibit nearly complete loss of foot processes, resulting in uniform swelling and widening, with podocytes adopting a simplified appearance characterized by linearized SD patterns, which we term "Pattern-1 retraction". In contrast, in the MCD group (B), although the foot processes were bluntly thickened and widened, they remained discernible. The region of lateral podocyte processes (RLPs) shows increased width but maintains a similar three-dimensional structure, presenting relatively tortuous SD patterns, which we classify as "Pattern-2 retraction". Scale bars = 20 μm and 1 μm (zoomed images). Abbreviations: S, sclerosis; CAP, capillary lumen; US, urinary space; CB, cell body; PP, primary process; FP, foot process.

Compared with MCD patients, FSGS patients had a significantly lower SD density (p < 0.05), wider PP (p < 0.05), and notably wider FPs (p < 0.0001), with the latter difference being more pronounced. Notably, in nonsclerotic FSGS glomeruli (Figure 7B) and podocytes located away from the sclerotic regions (Figure 8A), consistent PP simplification accompanied by the retraction and flattening of FPs was observed. This was accompanied by a transformation of the SD into a linear configuration, which we termed “pattern-1 retraction”. In contrast, at the MCD (Figure 7A, Figure 8B), the FPs exhibited widening and effacement but retained their three-dimensional integrity. This was concomitant with varying degrees of podocyte swelling and a characteristic meandering pattern of the SD, a phenomenon termed “pattern-2 retraction”.

These findings demonstrate that super-resolution imaging of ANGPTL4 and nephrin is a powerful clinical tool for assessing damage to the glomerular filtration barrier, highlighting the distinct pathological podocyte injury patterns in FSGS patients, who show more severe damage than MCD patients do, despite their similar clinical presentations.

## Discussion

**Figure 9:**
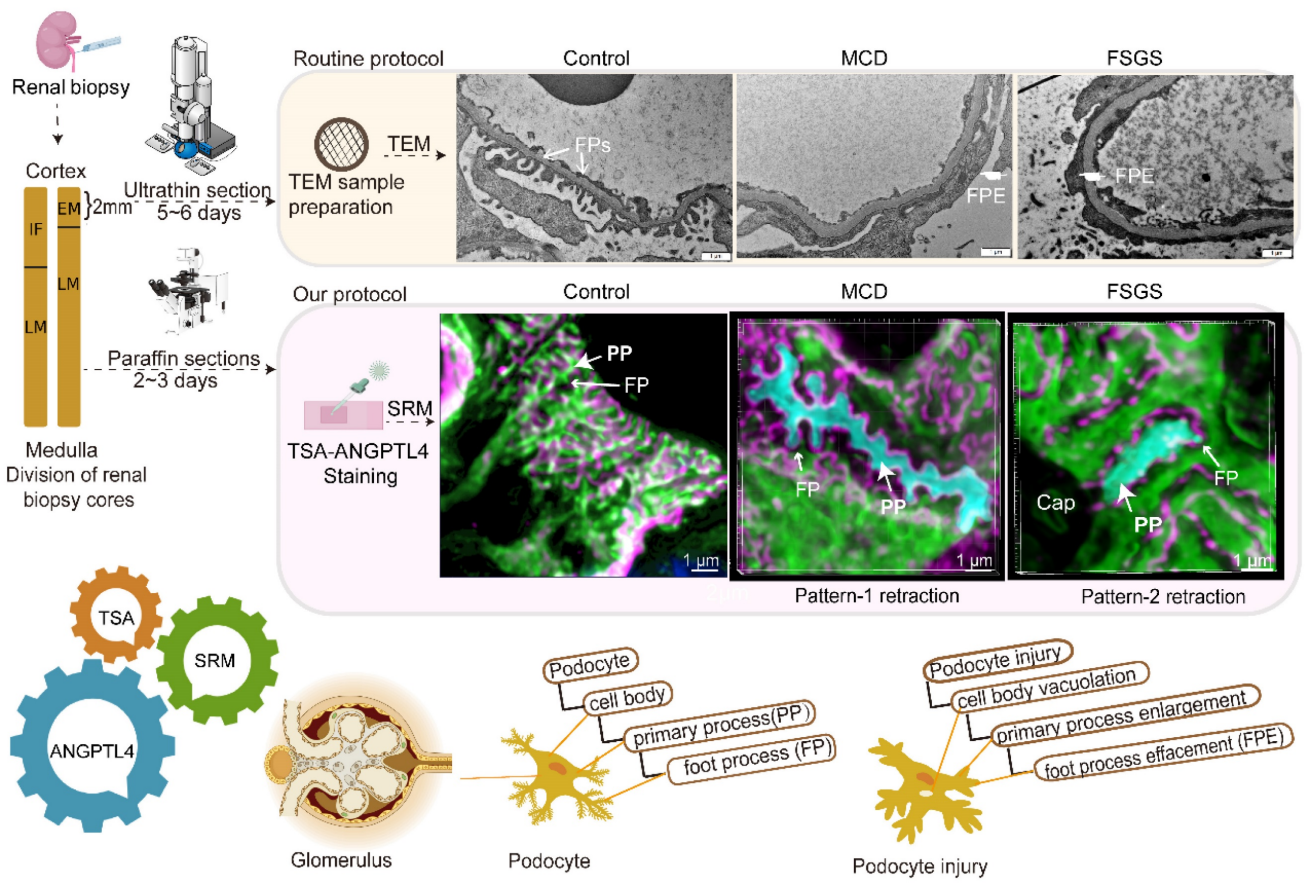
** A comparative study between the super-resolution examination of renal biopsy samples following ANGPTL4 staining and the traditional electron microscopy (EM) findings.** After renal biopsy, the tissue was divided, with a portion reserved for EM and the rest reserved for light microscopy (LM) and immunofluorescence (IF). Special handling is required for EM tissue, which allows for detailed examination of one or two glomeruli. A formalin-fixed paraffin-embedded (FFPE) section was subjected to ANGPTL4 staining via tyramine signal amplification (TSA) and imaged via super-resolution microscopy (SRM). SRM provides nanoscale insights into podocyte abnormalities, aiding in the detection of cellular vacuolization, thickening and swelling of primary processes, and foot process effacement. This approach transitions the assessment of podocyte injury from a 2D EM view to a 3D volumetric analysis, enhancing the accuracy of pathological diagnosis. Our method offers a potentially complementary or alternative strategy for the thorough evaluation of podocyte lesions in renal biopsy samples. This image was created with the help of the Fig-draw website, ID: RSUPS4444d.

In this study, we validated ANGPTL4 as a podocyte marker for observing and measuring podocyte morphology via a multifaceted approach that included analysis of fresh-frozen and FFPE tissues, along with immunoelectron microscopy and super-resolution techniques, to accurately map the molecular localization of ANGPTL4 *in situ*. We further developed a streamlined, robust imaging protocol (Figure 9) for clinical FFPE samples to evaluate the three-dimensional architecture of podocytes, primarily featuring optimized ANGPTL4 immunofluorescence staining and array microscopy, designed to satisfy clinical criteria for simplicity, sensitivity, field of view, and imaging speed. Using this method, we identified two distinct patterns of FP fusion in patients with MCD patients with FSGS who presented with massive proteinuria at onset.

ANGPTL4 is a glycoprotein of 45-65 kDa that is part of the ANGPTL family and is regulated by factors such as hypoxia and peroxisome proliferator activated receptor 31-33. Since its discovery two decades ago, ANGPTL4 has emerged as a multifaceted protein with key roles in energy balance, the inhibition of lipoprotein lipase, wound healing, blood vessel regulation, and potential involvement in cancer growth; ANGPTL4 is predominantly expressed in liver and adipose tissue and has individual variability 31, 33, 34. A decade ago, Clement *et al.* reported for the first time that podocytes secrete a hyposialylated form of ANGPTL4, which is upregulated and mediates proteinuria in MCD in animal models, marking a landmark discovery for the role of ANGPTL4 in the kidney 18. Subsequent studies have consistently revealed increased expression of ANGPTL4 in various renal pathologies, including diabetic nephropathy (DN), IgA nephropathy, and MN, which is consistent with our findings 21, 35-39.

Our study revealed that ANGPTL4 is not underexpressed in tumor-adjacent tissues, possibly because of several factors: 1. The increased secretion of podocytes may be influenced by a cytokine storm 26. 2. Both *in situ* hybridization and immunofluorescence confirmed that some glomerular endothelial cells in the peritumoral kidney are also a source of ANGPTL4 secretion, which may be related to tumor migration 25, 40. 3. The antibody we used is a polyclonal antibody, which, according to WB information, may target the C-terminal fibrinogen-like domain (C-ANGPTL4) rather than full-length ANGPTL4, possibly representing a degradation fragment of ANGPTL4 within the cell; 4. Compared with the frozen tissues used in other studies, FFPE tissues can better preserve the antigenicity of ANGPTL4. In contrast, the expression of ANGPTL4 in renal biopsy tissues from patients with interstitial nephritis is closer to the basal expression of human kidney tissue 18.

Clement's team discovered that among animal models of kidney disease, MCD demonstrated the most significant upregulation, beginning prior to the onset of proteinuria and reaching a peak shortly thereafter 18. In contrast, MN model exhibited a milder upregulation post-proteinuria, and the collapsing FSGS model showed no changes at all 20. In our research, which utilized human renal biopsy samples from individuals with primary nephrotic syndrome, we concentrated on the expression of ANGPTL4 in renal tissues post-massive proteinuria episodes. We could not track ANGPTL4 expression prior to disease onset but discovered a notable increase in ANGPTL4 at both the protein and RNA levels within one month of such episodes across various kidney pathologies. The observed increase in ANGPTL4 initially subsided after one month, yet protein expression remained detectable. We postulate that the sustained presence of ANGPTL4 could be attributed to two mechanisms: first, C-ANGPTL4, resulting from proteolytic cleavage of full-length ANGPTL4, which is detectable by the antibody; second, the significant secretion of ANGPTL4 by extrarenal sources such as skeletal muscle, liver, and adipose tissue under nephrotic conditions may lead to ANGPTL4 binding to podocyte receptors 18-20 in the kidneys. Additionally, endocytosis of ANGPTL4 via syndecan family proteins 20 could contribute to the sustained presence of this protein in the renal environment. These findings suggest that ANGPTL4 plays an important role throughout the entire course of the disease.

Interestingly, we also observed increased ANGPTL4 protein levels in FSGS patients, diverging from the findings of Clement's team in collapsing FSGS animal models. The divergence in our findings compared with those of Clement's team can be ascribed to a few key factors. Most notably, their animal models were developed using serum from patients with collapsing FSGS, an approach that failed when serum from patients with non-collapsing FSGS was used 41. Our study, on the other hand, included patients with non-collapsing FSGS, suggesting a fundamental difference in the pathogenesis between the two subtypes of FSGS 42, 43. This distinction in serum factors and their resulting podocyte damage may explain the variations in ANGPTL4 expression and the underlying disease mechanisms between collapsing and non-collapsing FSGS.

Renal pathology diagnosis is on the cusp of becoming faster and more accurate because of the ever-evolving technologies and advancements in other industries. Advanced microwave-assisted sample preparation has significantly halved the time required for electron microscopy sample preparation 44, enabling advanced laboratories such as Arkana to complete electron microscopy diagnosis within just 2 to 3 days, which benefits patients and clinicians by providing quicker insights into renal conditions, allowing for earlier intervention and potentially better outcomes. The advent of super-resolution microscopy technology offers new methods and perspectives for observing nanoscale structures. Sophisticated microscopic technologies have significantly bolstered our capacity to chart the intricate spatial interplay among diverse molecules. Unlike SYNO, which is specifically localized to FPs, ANGPTL4 enjoys a more expansive distribution across podocytes, permeating all subcellular compartments and extending from the cell body to the FPs. The omnipresent profile of ANGPTL4 paves the way for super-resolution imaging techniques to refine the morphometric analysis of podocytes, advancing our understanding of their structural nuances. In addition, a meticulous examination of the three-dimensional distribution of SYNO within healthy podocytes revealed that SYNO from different FPs is interconnected. Previous reports have indicated that SYNO primarily interacts with α-actinin-4, extending the actin filaments induced by α-actinin-4 and thereby regulating the actin cytoskeleton 45-47. The function of this interconnected form requires further investigation.

SIM is frequently used for observing proteins such as nephrin in SDs 12, 13, 48. In our study, it successfully captured the two-dimensional morphology of podocyte FPs but did not ideally visualize the tertiary structures of podocytes. Similarly, Kaufmann and Dobbie's comparative analysis revealed that SIM offers clear imaging for widely spaced and dense, z-axis extending structures, with the caveat that its performance is suboptimal for fine, dense three-dimensional networks 49. In summary, SIM may be less effective for resolving densely packed cellular ultrastructures such as podocytes. By incorporating ANGPTL4 staining, our study provided direct insight into the alterations in podocyte shapes when SDs are disrupted or straightened, addressing the issue raised by Pullman and Benzing regarding the difficulty in discerning the fusion of podocyte FPs when SD staining is suboptimal or when podocyte damage is severe 6, 8. There is an ongoing debate about whether MCD and FSGS represent different stages of a single disease or are two distinct conditions 50, 51. Thomas Benzing and his team used SD morphology and deep learning to analyze 22 pediatric MCD biopsy samples and one FSGS sample, finding distinct morphological differences between untreated patients and those receiving standard prednisone therapy 8. To eliminate the influence of glucocorticoids on podocyte morphology, we conducted a rigorous comparative study with newly diagnosed, treatment-naïve MCD and FSGS patients, all of whom presented with severe proteinuria of approximately 10 grams per day. Our analysis revealed that FSGS patients had more pronounced podocyte simplification, which is consistent with Weening and Wetzels' electron microscopy studies that reported wider FPs in FSGS patients 52. We also identified two patterns of FPE on the basis of morphological differences and quantifiable indicators of the tertiary structure of podocytes. This evidence suggests different responses of podocytes to injury in FSGS and MCD patients, indicating potential distinct disease processes. Despite the small sample size, our results highlight the value of super-resolution analysis of ANGPTL4 and nephrin in the study of podocyte and SD morphology across diseases. This method could be crucial for understanding the pathophysiology of renal diseases and for developing therapeutic strategies. Our method has several advantages. First, the ability to visualize podocyte subcompartments comprehensively positions ANGPTL4 as an ideal 'all-in-one' marker, despite the mechanisms of its renal function still not being fully elucidated. Second, our approach is compatible with routine FFPE samples, and we have developed a straightforward technique that uses conventional immunohistochemistry and ordinary light microscopy to measure podocyte morphological parameters—specifically, the width of primary processes—to assess podocyte injury comprehensively. This method is user friendly and suitable for widespread clinical application. This technique complements ultrastructural observations made with electron microscopy, enabling a comprehensive assessment of podocyte pathology in the kidney. Furthermore, slides processed with TSA can be stored for long durations without any loss in signal intensity, providing the necessary flexibility to accommodate various clinical requirements. This study has several limitations. First, it was based on a limited patient cohort, and further research encompassing larger sample sizes and a wider spectrum of clinical phenotypes is imperative to deepen our understanding of the podocyte injury response. Second, the development of a sophisticated automated image analysis workflow for the future is essential but not without its challenges. Owing to the high degree of interdigitation among podocytes, achieving high-precision segmentation at the single-cell level necessitates further enhancement of the z-axis resolution. Additionally, the sample must be sufficiently thick to encompass the complete podocyte structure, ensuring that the analysis captures the full extent of the cellular architecture.

## Conclusions

In summary, we validated ANGPTL4 as a novel and effective marker for analyzing podocytes via SRM. This method enables clear visualization of the three-dimensional structures of podocytes, providing a valuable tool for the morphological study of podocytes in both healthy and diseased conditions. By offering a comprehensive and detailed perspective on podocyte injury responses, this method is crucial for enhancing diagnostic precision and may inspire groundbreaking approaches for the management of a wide array of diseases.

## Supplementary Material

Supplementary figures.

Movie S1 3D volumetric super-resolution confocal imaging of podocytes stained with ANGPTL4 (cyan) and SD stained with nephrin (magenta) from a patient with minimal change disease (MCD).

Movie S2 3D volumetric super-resolution confocal imaging of podocytes stained with ANGPTL4 (cyan) and SD stained with nephrin (magenta) from a patient with primary focal segmental glomerulosclerosis (FSGS).

## Figures and Tables

**Table 1:** Clinical information for the primary MCD, MN, FSGS patients and normal controls

Parameters	MCD-NS	MCD-PR	MCD-CR	MN-NS	FSGS-NS	Controls
Number	7	8	4	8	5	5
Male/female	3/4	4/4	2/2	5/3	4/1	3/2
Age [yr]	33.7 ± 14.5	26.1 ± 9.7	23.8 ± 7.7	39.9 ± 20.4	30.8 ± 14.1	35.8 ± 16.3
Duration of disease [months]	1.6 ± 1.5	17.1 ± 11.4	36.9 ± 8.1	48.7 ± 24.9	5.7 ± 5.5	-
Urinary protein [g/d]	9.9 ± 6.0	1.6 ± 0.9	0.15 ± 0.08	9.3 ± 5.5	9.0 ± 3.9	-
Serum Albumin [g/L]	20.4 ± 6.9	27.3 ± 6.6	31.3 ± 9.5	20.4 ± 3.6	20.3 ± 4.5	46.5 ± 0.7
Serum Creatinine [μmol/L]	76.8 ±18.5	61.7 ± 21.0	71.0 ± 10.1	84.8 ± 44.1	69.2 ± 19.4	63.5 ± 11.3
Glucocorticoids treated/untreated	4/3	2/6	2/2	6/2	1/4	-

Values are means ± standard deviation; CR, complete remission; NS, nephrotic syndrome; PR, partial remission; FSGS, focal segmental glomerulosclerosis; MCD, minimal change disease; MN, membranous nephropathy.

**Table 2:** Clinical information for the patients and controls

Parameters	MCD	MN	FSGS	TIN	Donor	Normal adjacent tissues
Number	9	9	4	3	3	3
Male/female	5/4	5/4	2/2	2/1	1/2	2/1
Age [years]	37.0 ± 15.4	57.8 ± 9.78	48.5 ± 27.1	54.0 ± 15.8	38.3 ± 14.6	65.7 ± 4.5
Duration of disease [months]	13.3 ± 17.1	13.6 ± 15.6	22.5 ± 41.0	1.8 ± 1.3	-	5.7 ± 5.5
Duration of the current relapse [months]	7.7 ± 15.6	8.3 ± 15.2	21.6 ± 41.6	-	-	-
Urinary protein [g/d]	8.76 ± 4.37	8.12 ± 4.05	6.89 ± 4.58	1.38 ± 0.32	-	-
Serum Albumin [g/L]	21.4 ± 4.1	28.0 ± 5.4	28.9 ± 13.8	40.9 ± 6.3	41.7 ± 2.1	40 ± 1.8
Serum Creatinine [μmol/L]	105.4 ± 74.7	82.6 ± 29.6	165.1 ± 181.7	207.4 ± 147.5	72.4 ± 14.5	91.3 ± 17.2
Glucocorticoids treated/untreated before this relapse	2/7	1/8	1/4	0/4	-	-
Glucocorticoids treated/untreated for this relapse	0/9	0/9	0/4	0/3	-	-

Values are means ± standard deviation; FSGS, focal segmental glomerulosclerosis; MCD, minimal change disease; MN, membranous nephropathy; TIN, tubulointerstitial nephritis.

**Table 3:** Baseline characteristics

Patient	Urinary protein [g/day]	Age at biopsy [years]	Sex	Duration of disease [weeks]	Current therapy	Serum albumin [g/L]	Serum creatinine [μmol/L]	Diabetes	Follow-up
MCD1	10.66	41	male	3	none	21.7	76.6	no	glucocorticoid-sensitive
MCD2	8.65	25	female	3	none	18.9	79.3	no	glucocorticoid-resistant
MCD3	9.86	39	male	1	none	20.4	88	no	glucocorticoid-sensitive
FSGS1	11.72	27	male	3	Huangkui capsule	24.8	82.4	no	glucocorticoid-sensitive
FSGS2	9.72	23	male	2	Huangkui capsule	18.5	83	no	glucocorticoid-resistant
FSGS3	9.26	42	female	2	none	19.2	67.8	no	glucocorticoid-sensitive

## Abbreviations

ANGPTL4: angiopoietin-like 4
CAP: capillary lumen
CB: cell body
EM: electron microscopy
FFPE: formalin-fixed, paraffin-embedded
FIB/SEM: focused ion beam/scanning electron microscopy
FPE: foot process effacement
FP: foot process
FSGS: focal segmental glomerulosclerosis
KO: knockout
MCD: minimal change disease
MN: membranous nephropathy
PP: primary process
RLP: ridge-like prominence
SD: slit diaphragm
SIM: structured illumination microscopy
SRM: super-resolution microscopy
STED: stimulated emission depletion
SYNO: synaptopodin
TEM: transmission electron microscopy
TIN: tubulointerstitial nephritis
TSA: tyramide signal amplification
US: urinary space

## References

[1] Brinkkoetter PT, Ising C, Benzing T (2013). The role of the podocyte in albumin filtration. Nat Rev Nephrol.

[2] Florian Grahammer CS, Tobias B Huber (2013). The podocyte slit diaphragm-from a thin grey line to a complex signalling hub. Nat Rev Nephrol.

[3] Nagata M (2016). Podocyte injury and its consequences. Kidney Int.

[4] S O Bohman GJ, A B Bohlin, U Berg (1984). Foot process fusion and glomerular filtration rate in minimal change nephrotic syndrome. Kidney Int.

[5] Benzing T, Salant D (2021). Insights into glomerular filtration and albuminuria. New Engl J Med.

[6] Pullman JM (2019). New views of the glomerulus: advanced microscopy for advanced diagnosis. Front Med (Lausanne).

[7] Butt L, Unnersjo-Jess D, Hohne M, Edwards A, Binz-Lotter J, Reilly D (2020). A molecular mechanism explaining albuminuria in kidney disease. Nat Metab.

[8] Unnersjo-Jess D, Butt L, Hohne M, Sergei G, Fatehi A, Witasp A (2023). Deep learning-based segmentation and quantification of podocyte foot process morphology suggests differential patterns of foot process effacement across kidney pathologies. Kidney Int.

[9] Butt L, David Unnersjö-Jess (2022). Super-resolution imaging of the filtration barrier suggests a role for podocin R229Q in genetic predisposition to glomerular disease. J Am Soc Nephrol.

[10] Unnersjo-Jess D, Butt L, Hohne M, Witasp A, Kuhne L, Hoyer PF (2021). A fast and simple clearing and swelling protocol for 3D in-situ imaging of the kidney across scales. Kidney Int.

[11] Unnersjo-Jess D, Ramdedovic A, Butt L, Plagmann I, Hohne M, Hackl A (2023). Advanced optical imaging reveals preferred spatial orientation of podocyte processes along the axis of glomerular capillaries. Kidney Int.

[12] Siegerist F, Ribback S, Dombrowski F, Amann K, Zimmermann U, Endlich K (2017). Structured illumination microscopy and automatized image processing as a rapid diagnostic tool for podocyte effacement. Sci Rep.

[13] Pullman JM, Nylk J, Campbell EC, Gunn-Moore FJ, Prystowsky MB, Dholakia K (2016). Visualization of podocyte substructure with structured illumination microscopy (SIM): a new approach to nephrotic disease. Biomed Opt Express.

[14] Unnersjo-Jess D, Scott L, Blom H, Brismar H (2016). Super-resolution stimulated emission depletion imaging of slit diaphragm proteins in optically cleared kidney tissue. Kidney Int.

[15] Faul C, Donnelly M, Merscher-Gomez S, Chang YH, Franz S, Delfgaauw J (2008). The actin cytoskeleton of kidney podocytes is a direct target of the antiproteinuric effect of cyclosporine A. Nat Med.

[16] Agarwal S, Sudhini YR, Polat OK, Reiser J, Altintas MM (2021). Renal cell markers: lighthouses for managing renal diseases. Am J Physiol Renal Physiol.

[17] Ichimura K, Miyaki T, Kawasaki Y, Kinoshita M, Kakuta S, Sakai T (2019). Morphological processes of foot process effacement in puromycin aminonucleoside nephrosis revealed by FIB/SEM tomography. J Am Soc Nephrol.

[18] Clement LC, Avila-Casado C, Mace C, Soria E, Bakker WW, Kersten S (2011). Podocyte-secreted angiopoietin-like-4 mediates proteinuria in glucocorticoid-sensitive nephrotic syndrome. Nat Med.

[19] Clement LC, Mace C, Avila-Casado C, Joles JA, Kersten S, Chugh SS (2014). Circulating angiopoietin-like 4 links proteinuria with hypertriglyceridemia in nephrotic syndrome. Nat Med.

[20] Chugh SS, Clement LC (2023). "Idiopathic" minimal change nephrotic syndrome: a podocyte mystery nears the end. Am J Physiol Renal Physiol.

[21] Srivastava SP, Zhou H, Shenoi R, Morris M, Goedeke L, Rajendran BK (2023). Renal Angptl4 is a key fibrogenic molecule in progressive diabetic kidney disease. bioRxiv.

[22] Li Y, Xu Z, Deng H, Liu M, Lin X, Zhang M (2023). ANGPTL4 promotes nephrotic syndrome by downregulating podocyte expression of ACTN4 and podocin. Biochem Biophys Res Commun.

[23] Johnson KA (1995). Immunoelectron microscopy. Methods Cell Biol.

[24] Pasqualin C, Gannier F, Yu A, Benoist D, Findlay I, Bordy R (2022). Spiky: an imageJ plugin for data analysis of functional cardiac and cardiomyocyte studies. JImaging.

[25] Chaube B, Citrin KM, Sahraei M, Singh AK, de Urturi DS, Ding W (2023). Suppression of angiopoietin-like 4 reprograms endothelial cell metabolism and inhibits angiogenesis. Nat Commun.

[26] Del Nogal Avila M, Das R, Kharlyngdoh J, Molina-Jijon E, Donoro Blazquez H, Gambut S (2023). Cytokine storm-based mechanisms for extrapulmonary manifestations of SARS-CoV-2 infection. JCI Insight.

[27] Refaeli I, Hughes MR, Wong AK-W, Bissonnette MLZ, Roskelley CD, Wayne Vogl A (2020). Distinct functional requirements for podocalyxin in immature and mature podocytes reveal mechanisms of human kidney disease. Sci Rep.

[28] Naylor RW, Morais MRPT, Lennon R (2021). Complexities of the glomerular basement membrane. Nat Rev Nephrol.

[29] Pavenstädt H, Kriz W, Kretzler M (2003). Cell biology of the glomerular podocyte. Physiol Rev.

[30] Jonkman J, Brown CM, Wright GD, Anderson KI, North AJ (2020). Tutorial: guidance for quantitative confocal microscopy. Nat Protoc.

[31] Bouleti C, Monnot C, Germain S (2018). ANGPTL4, a multifaceted protein at the cross-talk between metabolism and cardiovascular disorders. Int J Cardiol.

[32] Ruscica M, Zimetti F, Adorni MP, Sirtori CR, Lupo MG, Ferri N (2020). Pharmacological aspects of ANGPTL3 and ANGPTL4 inhibitors: New therapeutic approaches for the treatment of atherogenic dyslipidemia. Pharmacol Res.

[33] Shuff S, Oyama Y, Walker L, Eckle T (2021). Circadian angiopoietin-like-4 as a novel therapy in cardiovascular disease. Trends Mol Med.

[34] Singh AK, Chaube B, Zhang X, Sun J, Citrin KM, Canfrán-Duque A (2021). Hepatocyte-specific suppression of ANGPTL4 improves obesity-associated diabetes and mitigates atherosclerosis in mice. J Clin Invest.

[35] Liu X (2014). Initial investigation into podocyte morphological alterations and the pathogenic role of angiopoietin-like protein 4 in minimal change disease. Master's thesis, Peking University.

[36] Ma J, Chen X, Li JS, Peng L, Wei SY, Zhao SL (2015). Upregulation of podocyte-secreted angiopoietin-like-4 in diabetic nephropathy. Endocrine.

[37] Li JS, Chen X, Peng L, Wei SY, Zhao SL, Diao TT (2015). Angiopoietin-like-4, a potential target of tacrolimus, predicts earlier podocyte injury in minimal change disease. PLoS One.

[38] Jia S, Peng X, Liang L, Zhang Y, Li M, Zhou Q (2020). The study of angptl4-modulated podocyte injury in IgA nephropathy. Front Physiol.

[39] Peng L, Ma J, Cui R, Chen X, Wei SY, Wei QJ (2014). The calcineurin inhibitor tacrolimus reduces proteinuria in membranous nephropathy accompanied by a decrease in angiopoietin-like-4. PLoS One.

[40] Verine J, Lehmann-Che J, Soliman H, Feugeas JP, Vidal JS, Mongiat-Artus P (2010). Determination of angptl4 mRNA as a diagnostic marker of primary and metastatic clear cell renal-cell carcinoma. PLoS One.

[41] Avila-Casado Mdel C, Perez-Torres I, Auron A, Soto V, Fortoul TI, Herrera-Acosta J (2004). Proteinuria in rats induced by serum from patients with collapsing glomerulopathy. Kidney Int.

[42] De Vriese AS, Wetzels JF, Glassock RJ, Sethi S, Fervenza FC (2021). Therapeutic trials in adult FSGS: lessons learned and the road forward. Nat Rev Nephrol.

[43] Meliambro K, He JC, Campbell KN (2024). Podocyte-targeted therapies - progress and future directions. Nat Rev Nephrol.

[44] Zechmann B, Zellnig G (2009). Microwave-assisted rapid plant sample preparation for transmission electron microscopy. J Microsc.

[45] Asanuma K, Yanagida-Asanuma E, Faul C, Tomino Y, Kim K, Mundel P (2006). Synaptopodin orchestrates actin organization and cell motility via regulation of RhoA signalling. Nat Cell Biol.

[46] Ning L, Suleiman HY, Miner JH (2020). Synaptopodin is dispensable for normal podocyte homeostasis but is protective in the context of acute podocyte injury. J Am Soc Nephrol.

[47] Kannan N, Tang VW (2015). Synaptopodin couples epithelial contractility to alpha-actinin-4-dependent junction maturation. J Cell Biol.

[48] Sheppard CJR (2021). Structured illumination microscopy and image scanning microscopy: a review and comparison of imaging properties. Philos Trans A Math Phys Eng Sci.

[49] Wegel E, Göhler A, Lagerholm BC, Wainman A, Uphoff S, Kaufmann R (2016). Imaging cellular structures in super-resolution with SIM, STED and Localisation Microscopy: A practical comparison. Sci Rep.

[50] Maas RJ, Deegens JK, Smeets B, Moeller MJ, Wetzels JF (2016). Minimal change disease and idiopathic FSGS: manifestations of the same disease. Nat Rev Nephrol.

[51] Ozeki T, Maruyama S, Imasawa T, Kawaguchi T, Kitamura H, Kadomura M (2021). Clinical manifestations of focal segmental glomerulosclerosis in Japan from the Japan Renal Biopsy Registry: age stratification and comparison with minimal change disease. Sci Rep.

[52] Deegens JK, Dijkman HB, Borm GF, Steenbergen EJ, van den Berg JG, Weening JJ (2008). Podocyte foot process effacement as a diagnostic tool in focal segmental glomerulosclerosis. Kidney Int.

