# Supplementary material for: Advancing the clinical assessment of glomerular podocyte pathology in kidney biopsies via super-resolution microscopy and angiopoietin-like 4 staining
Source: Theranostics. 2025 Jan 1;15(3):784–803. doi: 10.7150/thno.101498 (PMC11700855; doi:10.7150/thno.101498)
Supplement: Supplementary file 1 — Supplementary figures. [file thnov15p0784s1.pdf]

Supplementary Materials

for

Advancing the clinical assessment of glomerular podocyte pathology in kidney biopsies via super-resolution microscopy and angiopoietin-like 4 staining

Xiaojing Liu, Suxia Wang, Gang Liu, Yan Wang, Shunlai Shang, Guming Zou, Shimin Jiang, Xuliang Wang, Li Yang✉, Wenge Li✉

This supplementary file has 8 figures.

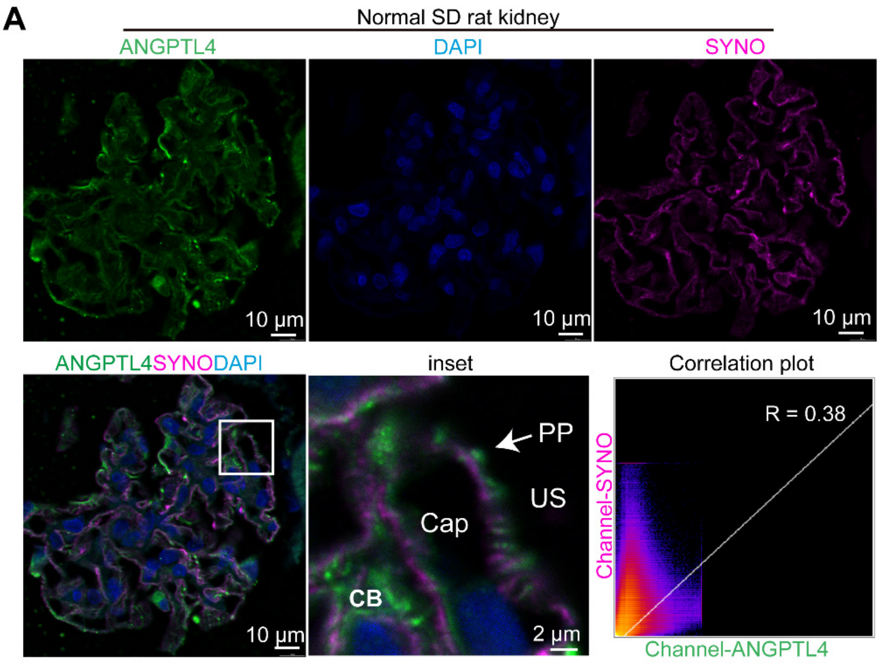

Figure S1. Immunofluorescence validation of the ANGPTL4 antibody in the kidneys of Sprague–Dawley (SD) rats. (A) Images were captured via a Leica STELLARIS confocal microscope (Buffalo Grove, IL, USA) at 40× magnification. Formalin-fixed, paraffin-embedded kidney tissues from normal SD rats were stained with 405-labeled DAPI, DyLight488-labeled ANGPTL4, and Alexa Fluor 649-labeled synaptopodin (SYNO). Scale bars represent 10 μm and 2 μm (for zoomed images). Immunofluorescence images were analyzed for ANGPTL4 and SYNO colocalization via the Coloc2 ImageJ plugin. The 2D intensity histogram indicates that 38% of these proteins colocalized.

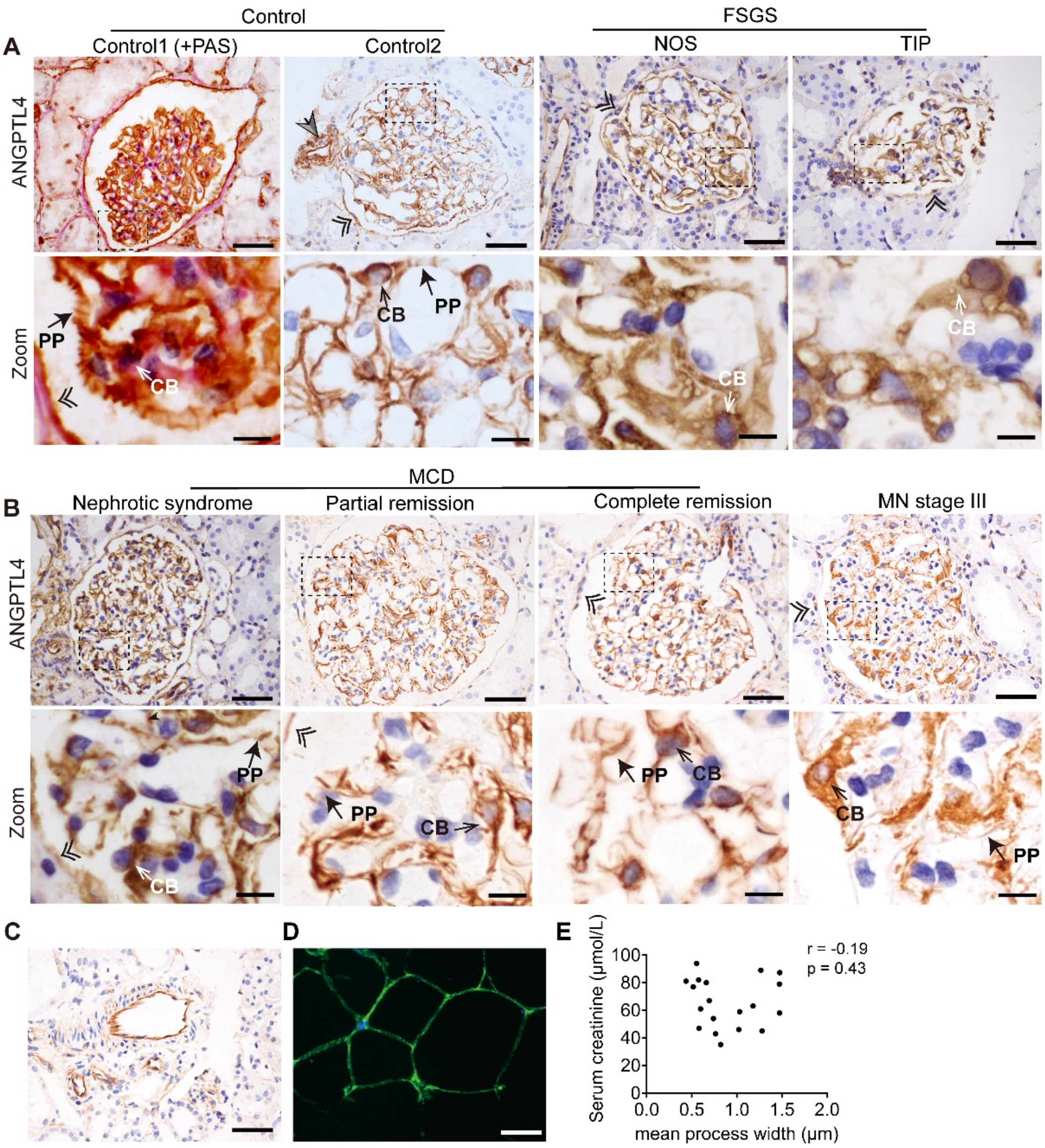

Figure S2. Immunohistochemistry revealed notable ANGPTL4 expression in podocytes across control and various disease conditions. ANGPTL4 is widely distributed in podocytes (arrows), parietal epithelial cells (double arrows), and vascular endothelium (gray arrows) in peritumoral renal tissue, healthy donor kidneys, focal segmental glomerulosclerosis (FSGS) not otherwise specified (NOS) and tip variants **(A)**, various stages of minimal change disease, and membranous nephropathy **(B)**. **(C-D)** High expression of ANGPTL4 is observed in the endothelium of interstitial blood vessels and fat. Scale bars = 50 μm and 10 μm (zoomed images). **(E)** Spearman

correlation analysis of the relationship between the mean process width and the serum creatinine level in 19 MCD patients revealed no significant correlation. The Spearman correlation coefficient and exact p value are given. Abbreviations: CAP, capillary lumen; US, urinary space; CB, cell body; PP, primary process; FP, foot process.

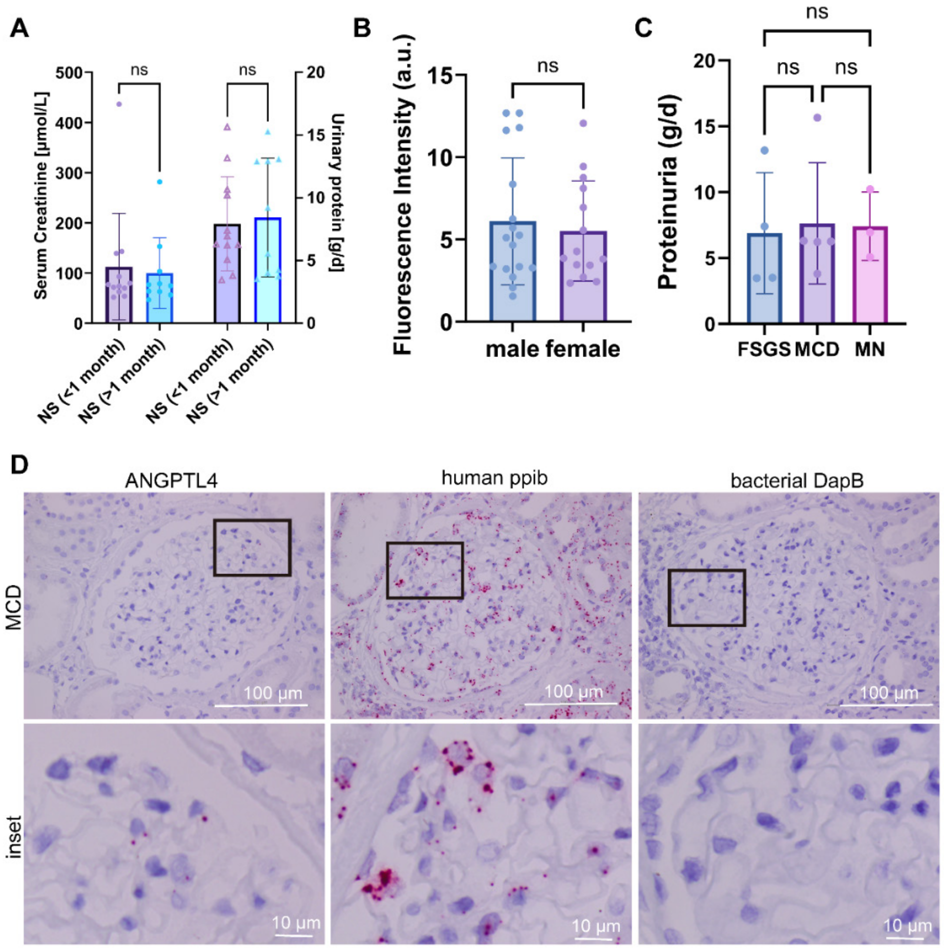

Figure S3. Comparative quantitative analysis of sex differences in ANGPTL4 immunofluorescence and quality control of ANGPTL4 in situ hybridization. (A) There were no statistically significant differences in proteinuria or creatinine levels at the time of renal biopsy between the two groups of patients shown in Figure 3 (those who had renal biopsies within 1 month after relapse and those after 1 month). **(B)** There were no differences in the degree of immunofluorescence quantified by sex. **(C)** There

was no statistically significant difference in proteinuria among the MCD, FSGS, and MN groups who underwent biopsies less than one month after relapse. The data are presented as the means ± standard deviations. Statistical significance was analyzed via one-way ANOVA. ns, not significant. **(D)** For quality control of in situ hybridization, human ppib served as a positive control, and bacterial DapB served as a negative control. Bars = 100 μm, inset 10 μm. Abbreviations: MCD, minimal change disease; NS, nephrotic syndrome.

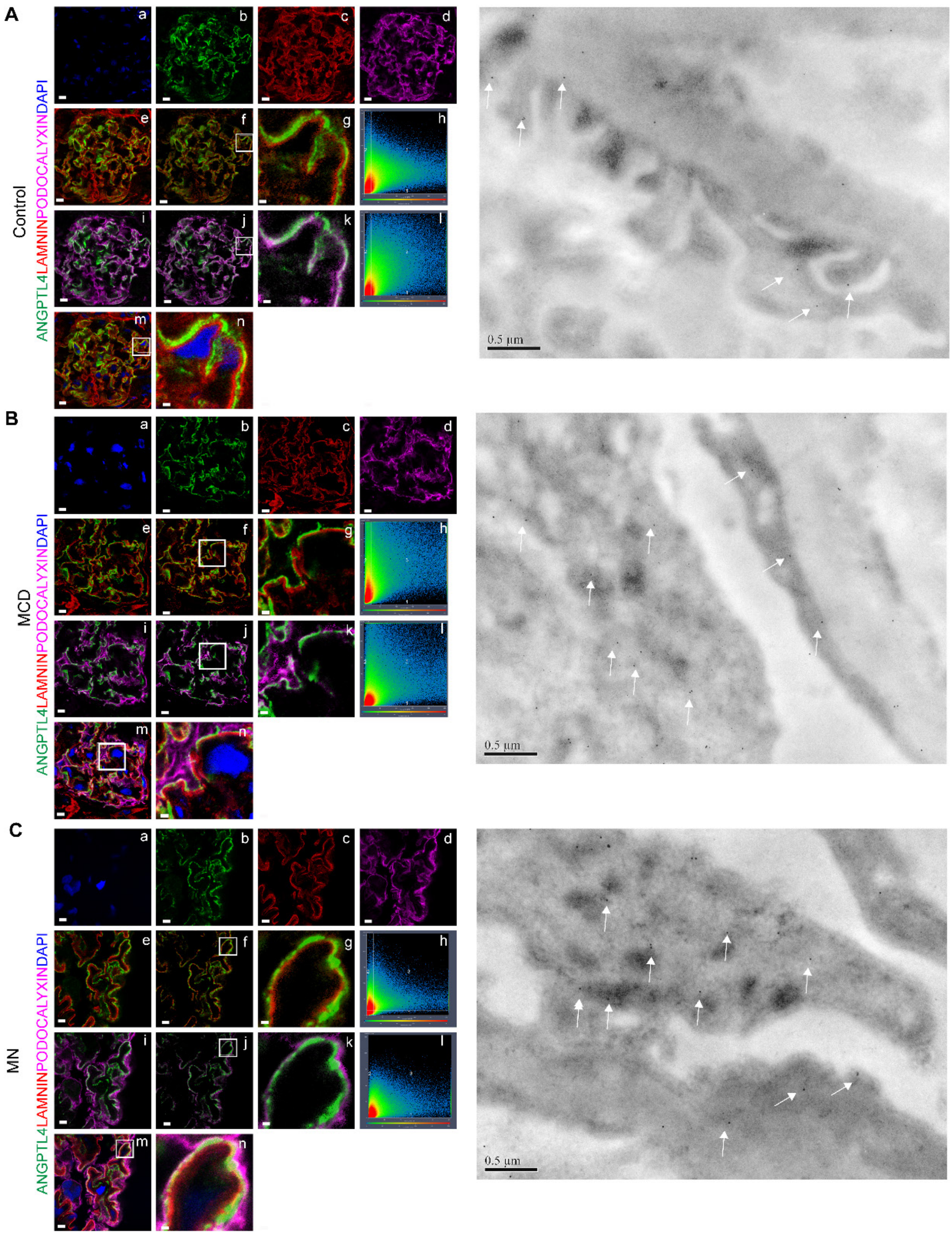

Figure S4. ANGPTL4 subcellular localization in podocytes from control, MCD, and MN patients by confocal and immunoelectron microscopy. All the confocal images were acquired via a Zeiss LSM 780 META instrument equipped with a 100×/1.40 Oil DIC M27 oil-immersion objective. Freshly frozen peritumoral kidney **(A)**, MCD **(B)**, and MN **(C)** tissues were stained with 405 nm-labeled DAPIa,

DyLight488-labeled ANGPTL4b, Cy3-labeled lamininc, and Alexa Fluor 649-labeled podocalyxind. The ANGPTL4 signal partially colocalized with laminin in G and podocalyxin in (k). (h-l) 2D frequency scatterplot of the ANGPTL4 and laminin/podocalyxin intensity distributions. (m) Merged image of ANGPTL4, laminin, podocalyxin and DAPI. The squares in figures f, j, and m are magnified and shown in g, k, and n, respectively. Bars =10 µm and 1 µm (zoomed images). Along the capillary wall, ANGPTL4 signals are distributed exterior to laminin signals and interior to podocalyxin signals. Immunogold particles were observed in the podocyte CB, PPs, and individual FPs in both the control samples and the samples with effaced FPs in the MCD and MN groups. Bars = 0.5 µm.

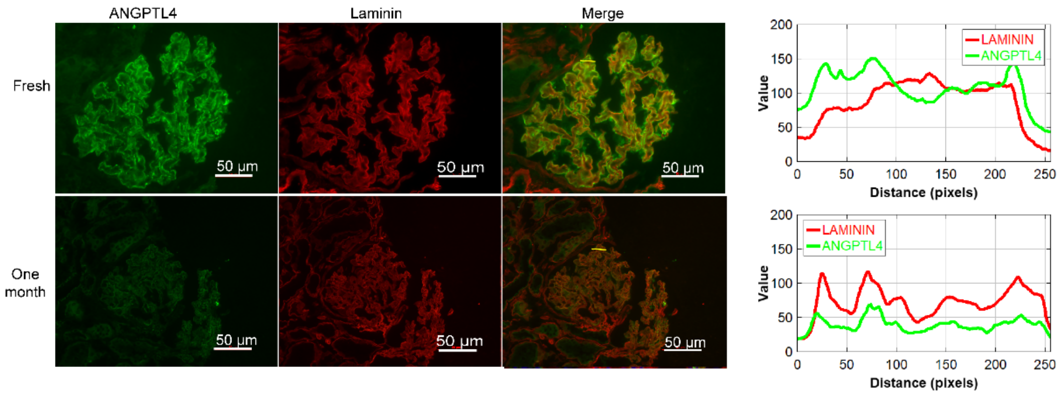

Figure S5. Comparative analysis of ANGPTL4 staining in fresh frozen versus long-term preserved renal tissue. The upper and lower rows display images of fresh frozen peritumoral renal tissue and tissue frozen after storage at -80 °C for one month, respectively, both subjected to dual immunofluorescence staining for ANGPTL4 (green) and laminin (red). On the rightmost side of each row, the fluorescence intensity profile along the yellow line is presented, illustrating the comparison between ANGPTL4 signals and laminin signals. The staining intensity for both ANGPTL4 and

laminin was comparable in fresh tissue; however, in long-term stored tissue, the ANGPTL4 signal intensity notably decreased relative to that of laminin, reaching approximately half of the laminin peak intensity. Scale bars = 50 μm.

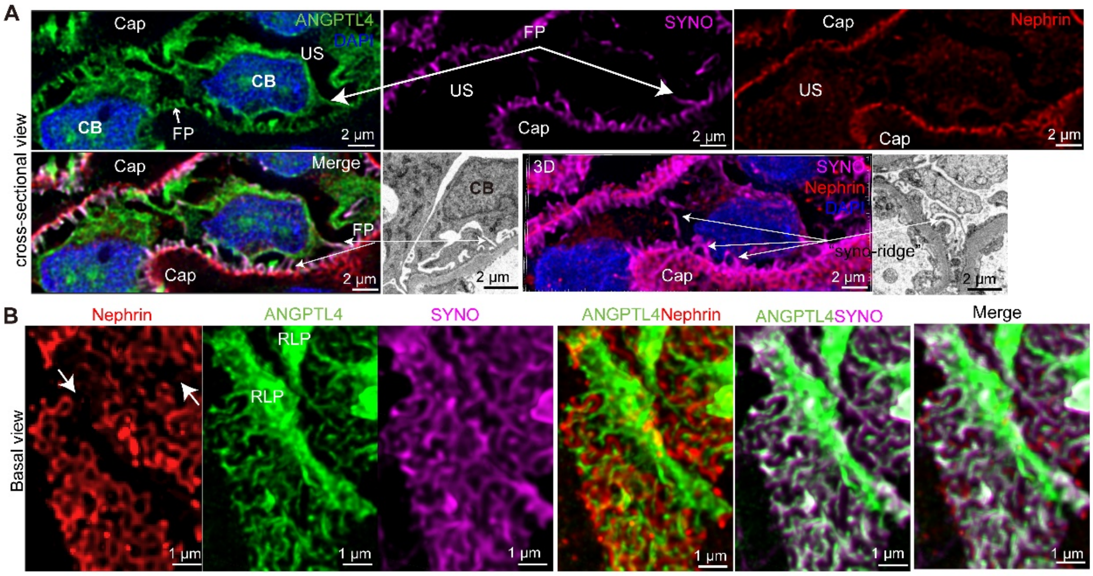

Figure S6. Super-resolution imaging of ANGPTL4 distribution in podocyte cross-sections and basal views. All images were captured and processed via a STELLARIS 8 FALCON system equipped with a 100×/1.4 numerical aperture oil immersion objective lens (pinhole set at 0.7 Airy Units). Three-micron-thick sections of donor kidney tissue were immunostained for ANGPTL4 with DyLight 488 (green), synaptopodin (SYNO) with Alexa Fluor 647 (magenta), and nephrin with CY3 (red) and counterstained with DAPI (blue). **(A)** The top row displays cross-sectional images from a single slice within a Z-stack or 3D reconstruction alongside electron micrographs. Scale bars = 2 μm. **(B)** The distribution characteristics of the three markers on the vascular basal side are shown. Scale bars = 1 μm. Abbreviations: CAP, capillary lumen; US, urinary space; CB, cell body; PP, primary process; FP, foot process.

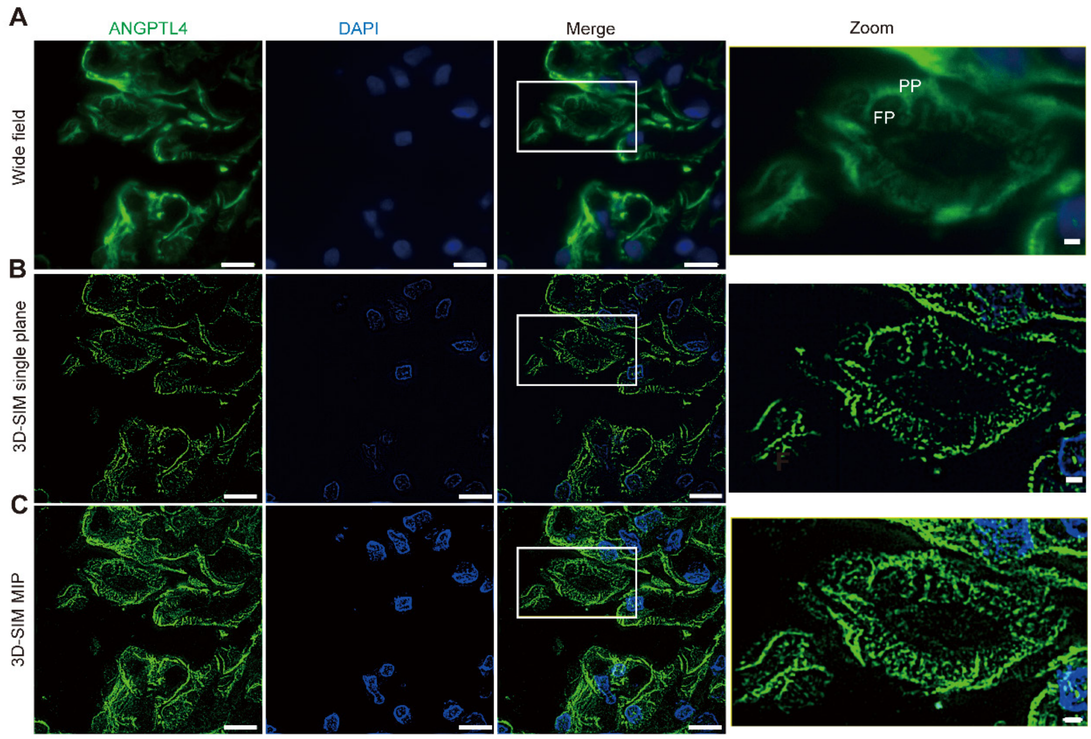

Figure S7. Wide-field (WF) and structured illumination microscopy (SIM) images of ANGPTL4-stained glomeruli from an FSGS patient. Comparison between wide-field microscopy image **(A)** and SIM **(B, C)** images of an "en face" view of the podocyte morphology from a patient diagnosed with cellular FSGS. All the images were stained for ANGPTL4 with DyLight-488 and acquired via a Nikon N-SIM S microscope with a 63×/1.4 NA oil objective. The micrographs in **(A)** show a single frame of the original fluorescence-WF image of podocytes prior to 3D-SIM reconstruction **(B-C)**. The staining pattern was linear, and podocyte primary processes (PPs) and secondary processes (FPs) were observed. The SIM image **(B)** and the maximum intensity projections of ~2-μm-thick Z-stacks **(C)** both depict podocyte structures, yet they exhibit a loss of continuity. The right column shows zoomed images of the areas indicated by a white square box. Bars = 10 μm and 2 μm (zoomed images).

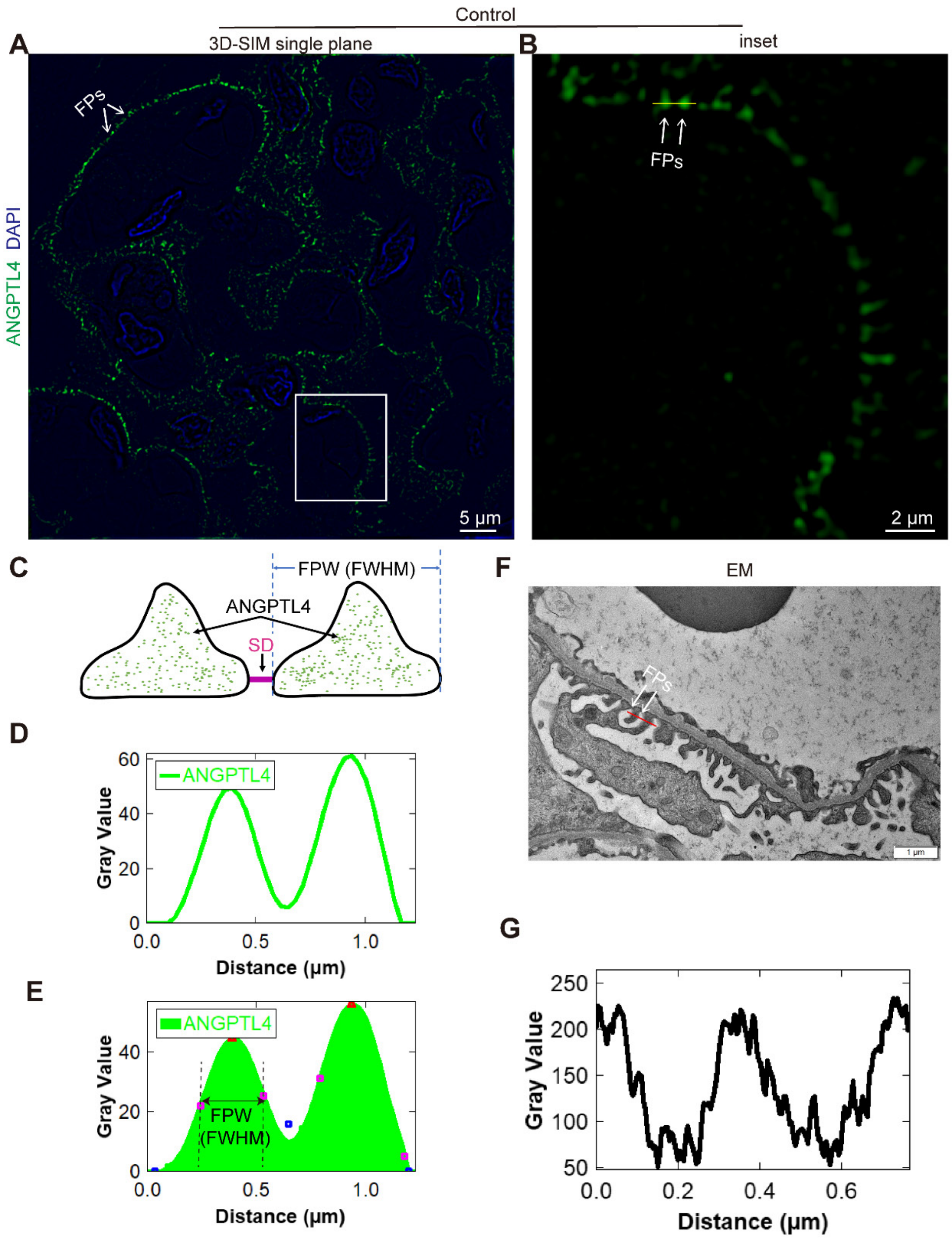

Figure S8. Structured illumination microscopy (SIM) and electron microscopy (EM) images of ANGPTL4-stained glomeruli from a healthy control. (A-B) ANGPTL4 in the control sample was stained with DyLight 488 (green) and DAPI (blue). For SIM imaging, a Nikon N-SIM S microscope with a 63×/1.4 NA oil objective was used. A higher magnification image corresponding to the squares in **(A)** is shown

in **(B)**. Bars = 10 µm **(A)**; 2 µm in **(B)**. **(A)** Cross-sectional view of foot process (FP) morphology in samples from a healthy control showing individual FPs (white arrows). **(C)** Schematic representation of the glomerular filtration barrier and localization of ANGPTL4 inside the foot process. **(D)** Corresponding intensity profile plots of the red lines **(B)**. **(E)** Line profile of the line in the SIM image of the control **(B)**, including a fit of the profile (black line) with several Gaussians (colorful dots) for determining the positions of the FPs. The foot process width (FPW) was measured as the full width at half maximum (FWHM) of multiple ANGPTL4 profiles. **(F)** EM image of glomeruli from the same patients shown for comparison. Bars = 1 µm. **(G)** Line profiles of the line in the EM image of the control **(F)**.
